# Donor-strength tuning enables a high-voltage all-p-type polymer cathode with fast redox kinetics for wide-temperature sodium dual-ion batteries

**DOI:** 10.1039/d6sc05745d

**Published:** 2026-08-28

**Authors:** Shuai Liu, Puiki Leung, Linyang Li, Yong Zuo, Lei Wei, Frank C. Walsh, Xun Zhu, Tianshou Zhao, Qiang Liao

**Affiliations:** a Chongqing University, Key Laboratory of Low-grade Energy Utilization Technologies and Systems, MOE Chongqing 400030 China P.leung@cqu.edu.cn; b Southern University of Science and Technology, Department of Mechanical and Energy Engineering Shenzhen 518055 China weil@sustech.edu.cn zhaots@sustech.edu.cn; c Electrochemical Engineering Laboratory, Department of Mechanical Engineering, Faculty of Engineering and Physical Sciences, University of Southampton SO17 1BJ UK

## Abstract

Sodium-ion batteries have emerged as a sustainable alternative to lithium-ion batteries, supported by the wide availability and low cost of sodium resources. However, many reported cathodes suffer from low redox potentials, sluggish kinetics, limited cycling stability and temperature sensitivity. Herein, we employed a donor-strength-tuning strategy to design and synthesize an all-donor conjugated polymer cathode, TPPAPZ, *via* cross-coupling polymerization of a moderate donor (*N*_1_,*N*_1_,*N*_4_,*N*_4_-tetrakis(4-bromophenyl)benzene-1,4-diamine, TPPA-4Br) with a strong donor (5,10-dihydrophenazine, PZ). The combination of two electronically differentiated donor units modulates the frontier electronic structure while maintaining a rigid conjugated backbone, providing a favorable electronic structure for charge transport. The TPPAPZ cathode delivers a high average discharge potential of 3.4 V, exceptional rate capability (128 mAh g^−1^ at 10 A g^−1^) and ultra-long cycling stability (50 000 cycles). Notably, it exhibits robust performance across a wide temperature range from −30 °C (118 mAh g^−1^) to 50 °C (160 mAh g^−1^). In full cells, TPPAPZ achieves ∼159 mAh g^−1^. This all-donor polymer design strategy provides a transferable route to mitigate the voltage-capacity trade-off in organic electrodes, enabling the development of long-cycle and wide-temperature sodium dual-ion batteries.

## Abstract 2

A donor-strength-tuned all-p-type polymer cathode enables stable sodium dual-ion battery operation from −30 to 50 °C.

## Graphical abstract

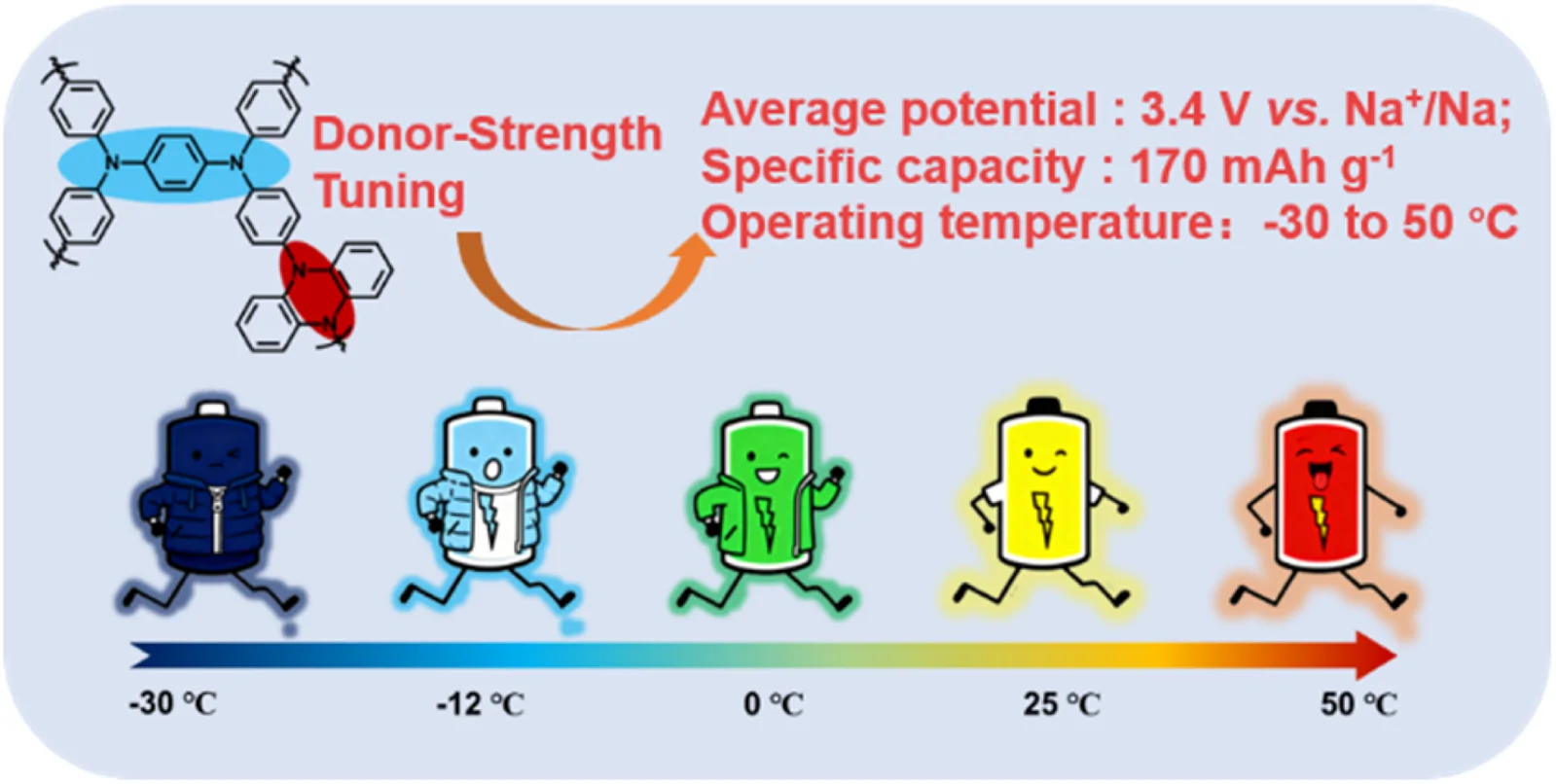

## 1 Introduction

The growing demand for large-scale energy storage in applications, such as grid storage and electric vehicles, has driven intensive research into sustainable and low-cost battery technologies.^1,2^ Lithium-ion batteries (LIBs) have dominated the market for decades, but their wider deployment is constrained by the scarcity and uneven distribution of lithium resources, which continue to raise concerns over cost and supply security.^3,4^ Sodium-based batteries have therefore emerged as attractive alternatives owing to the abundance and low cost of sodium, as well as its electrochemical similarity to lithium.^5–7^ Among them, sodium dual-ion batteries have attracted particular interest due to their ability to combine the low cost of sodium with high-voltage operation.^8^ However, the development of high-performance sodium dual-ion batteries remains restricted by the lack of suitable cathode materials.^6^ An ideal cathode must simultaneously deliver high redox potential, fast kinetics, excellent discharge capacity, long cycling stability and reliable operation over a wide temperature range.^9,10^

Organic conjugated polymers are promising cathode candidates due to their structural tunability, tailorable redox properties and environmental benignity.^11–14^ They also offer a possible route to reduce reliance on scarce inorganic cathode materials.^15–17^ Among them, p-type conjugated polymers based on electron-donating units are of particular interest.^18^ Their redox reactions involve the oxidation of electron-rich moieties such as nitrogen-containing aromatics, which typically occur at relatively high potentials.^19^ However, a major limitation of p-type polymers is their relatively low capacity.^20^ Previous works have attempted to address this issue by combining p-type donor units with n-type acceptor units to construct donor–acceptor (D–A) polymers, which can enhance the theoretical capacity of the cathode.^21–23^ But the introduction of n-type functional groups often reduces the average discharge potential and thus limits the overall energy density.^24,25^ For example, *Chen et al.* reported a polymer derived from electron-rich phenazine and electron-deficient anthraquinone, in which the n-type discharge plateaus are located at 1.6 V and 2.4 V, while the p-type discharge plateaus appear at 2.9 V and 3.0 V. Although this material delivers a discharge capacity of 270 mAh g^−1^, the introduction of the low-potential n-type anthraquinone units significantly lowers the overall average discharge voltage, thereby limiting its practical utility as a cathode material.^26^ In addition, many organic cathodes continue to suffer from insufficient intrinsic conductivity, poor structural stability during long-term redox cycling and significant performance decay under extreme temperatures, such as <0 °C or >40 °C. Together, these issues continue to hinder the development of high-performance organic cathodes for sodium-based batteries.

A key challenge is therefore to regulate the electronic structure of the conjugated backbone while retaining the high redox potential characteristic of p-type chemistry. Donor–donor (D–D) conjugated polymers, which link two or more electron-donating units without introducing n-type acceptors, have recently emerged as a promising alternative.^27^ Unlike D–A polymers, which rely on donor–acceptor push–pull interactions and often sacrifice voltage when acceptor units are introduced, D–D polymers retain a fully electron-rich backbone and therefore better preserve the high-potential character of p-type redox chemistry.^28–30^ However, the further development of D–D polymers requires more than simply combining donor units.^31^ A more effective design should introduce purposeful electronic differentiation within an all-donor framework.^32^ This enables the regulation of charge distribution, redox-site utilization, and carrier transport without relying on conventional acceptor units. When donor units with different electron-donating strengths are integrated into one conjugated backbone, the electronic structure of the polymer can still be effectively tuned without introducing a conventional acceptor unit.^33^ This creates an electronically differentiated all-donor backbone that offers a useful route to regulate frontier orbital distribution, charge delocalization and redox-site utilization while maintaining an all-p-type framework.^34^ From a mechanistic perspective, such donor-strength regulation can promote intramolecular charge redistribution and facilitate anion-compensated p-type redox processes, while also supporting fast charge transfer and structural stability.^35^ Thus, it offers an effective strategy for mitigating the usual voltage-capacity trade-off in organic cathodes while maintaining a purely donor-based polymer.^35^

Herein, we report a donor-strength-tuning molecular design strategy to construct an all-p-type conjugated polymer cathode, denoted TPPAPZ. This polymer integrates two donor units with distinct electron-donating strengths: *N*,*N*,*N*′,*N*′-tetraphenyl-*p*-phenylenediamine (TPPA) as a moderate electron donor, and 5,10-dihydrophenazine (PZ) as a strong electron donor. TPPAPZ integrates the advantages of both units. TPPA contributes excellent redox stability and high hole mobility. PZ provides a high discharge capacity and a rigid planar conjugated backbone. This rigid structure facilitates π–π stacking and interchain charge transport. Their combination generates an electronically differentiated all-donor backbone with optimized frontier orbital distribution, enhanced redox activity and improved structural stability.^36^ This donor-strength-tuning backbone is expected to regulate the electronic structure in a way that preserves the high-voltage character of p-type chemistry while enabling efficient charge transport and higher redox-site accessibility.^37^

In Na half-cells, TPPAPZ delivers an average discharge potential of 3.4 V and an energy density of 578 Wh kg^−1^ (based on the cathode active material). Remarkably, it retains 80% of its capacity (108 mAh g^−1^) after 50 000 cycles at 10 A g^−1^. It also maintains strong electrochemical performance over a wide temperature range, delivering 118 mAh g^−1^ at −30 °C, 136 mAh g^−1^ at 0 °C and 160 mAh g^−1^ at 50 °C under the respective testing conditions. When paired with hard-carbon anodes in full cells, it delivers 119 mAh g^−1^ over 2000 cycles. These results position TPPAPZ among the most competitive organic cathodes reported to date. More importantly, this work demonstrates that regulating electronic structure within an all-donor polymer backbone provides an effective and generalizable strategy for developing high-voltage, high-capacity, long-life and wide-temperature sodium dual-ion batteries.^38^

## 2 Results and discussion

### 2.1. Synthesis and characterization

**Fig. 1:**
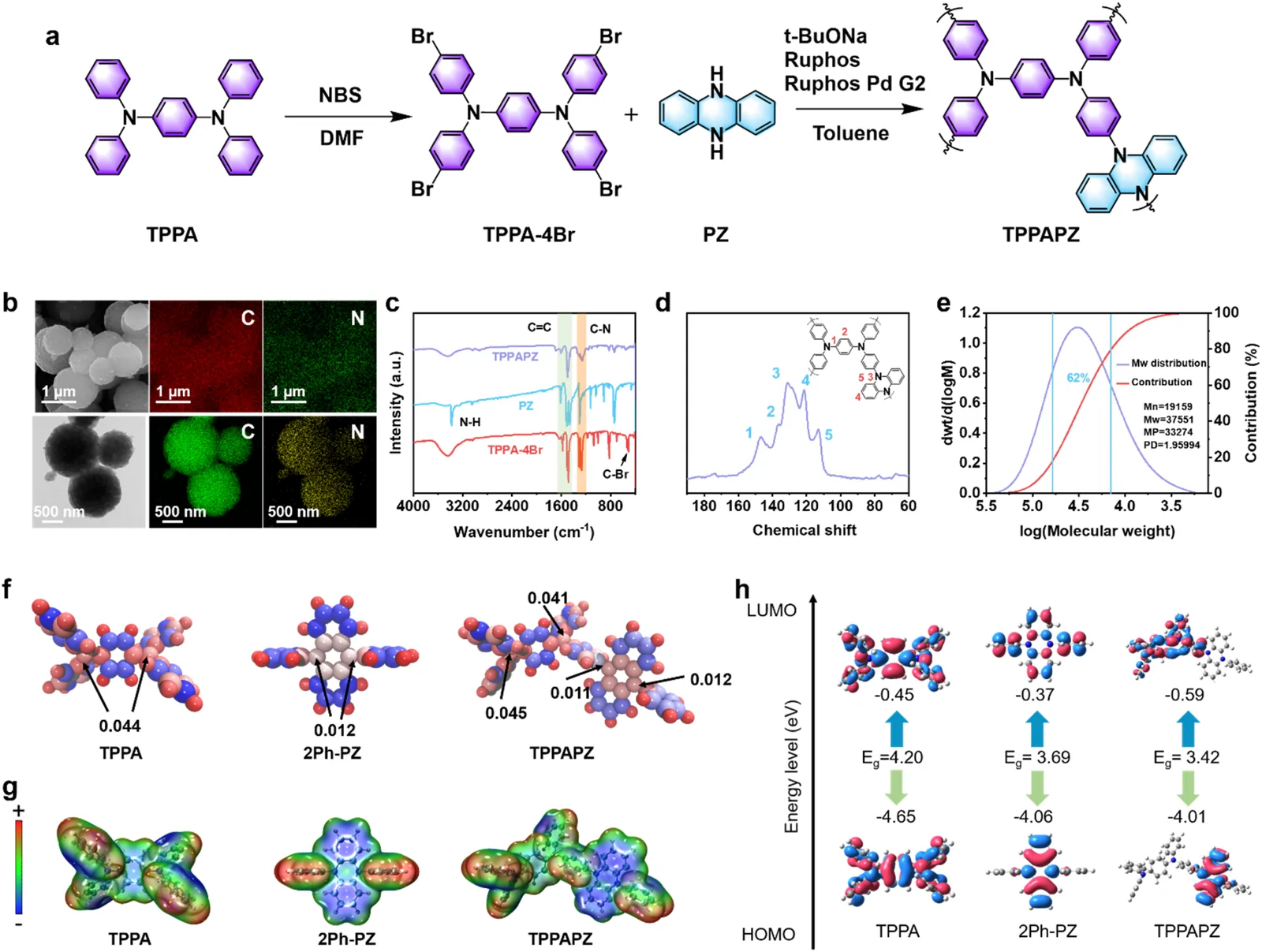
(a) Synthetic route to TPPAPZ. (b) TEM and SEM images with EDS elemental mapping of TPPAPZ. (c) FTIR spectra of TPPA-Br, PZ, and TPPAPZ. (d) Solid-state ^13^C NMR spectrum of TPPAPZ. (e) GPC trace of TPPAPZ. (f) ADCH charge distribution. (g) Electrostatic potential maps. (h) HOMO–LUMO energy levels and bandgap values.

The synthesis of TPPAPZ is illustrated in Fig. 1a. The detailed synthetic procedure and characterization data for *N*_1_,*N*_1_,*N*_4_,*N*_4_-tetrakis(4-bromophenyl) benzene-1,4-diamine (TPPA–4Br) are provided in the SI (Fig. S1–S4). TPPAPZ was obtained in high yield (91%) *via* Pd-catalyzed Buchwald-Hartwig C–N cross-coupling, which effectively links the TPPA and 5,10-dihydrophenazine (PZ) units (Fig. S5). The resulting gray powder exhibited negligible solubility in most common organic solvents (Fig. S6), a feature that contributes to its long-term cycling stability in organic electrolytes. Morphological characterization was conducted using transmission electron microscopy (TEM) and scanning electron microscopy (SEM) (Fig. S7 and S8). TPPAPZ adopted a distinctive micron-sized spherical architecture, contrasting with the typically amorphous nature of many conjugated polymers. Energy-dispersive X-ray spectroscopy (EDS) mapping confirmed uniform distribution of C and N elements throughout the spheres (Fig. 1b). Fourier-transform infrared (FTIR) spectroscopy confirmed the successful Buchwald-Hartwig coupling reaction (Fig. 1c). The characteristic C–Br stretching peak from TPPA–4Br and the N–H signal from PZ were both absent in the TPPAPZ spectrum, indicating complete consumption of the reactants and formation of the target polymer. X-ray photoelectron spectroscopy (XPS) further confirmed the successful coupling (Fig. S9 and S10). The C 1s spectrum showed only C=C and C–N peaks with no detectable C–Br signal, while the N 1s spectrum displayed solely C–N contributions. Solid-state ^13^C NMR (Fig. 1d) showed five distinct peaks, matching the five inequivalent carbon environments of TPPAPZ. X-ray diffraction (XRD) analysis showed that polymerization resulted in an amorphous TPPAPZ structure with broad, diffuse diffraction features, with the loss of crystallinity attributed to increased chain length and structural complexity (Fig. S11). Gel permeation chromatography (GPC) was performed to evaluate the molecular weight and molecular weight distribution of TPPAPZ (Fig. 1e). Owing to the poor solubility of TPPAPZ in conventional organic solvents, high-temperature trimethylbenzene was selected as the solvent for GPC measurements. The molecular weight distribution of TPPAPZ was primarily centered between 14 and 61 kDa, accounting for 63% of the total weight fraction. The calculated number-average molecular weight (*M*_n_) was 19 kDa and the weight-average molecular weight (*M*_w_) was 38 kDa, yielding a polydispersity index (PDI) of 2.0. Taken together, these results support the successful synthesis of TPPAPZ.

To theoretically understand the electronic structure, density functional theory (DFT) was performed on TPPA, 5,10-diphenyl-5,10-dihydrophenazine (2Ph-PZ) and the polymer TPPAPZ. To faithfully simulate the chemical environment of the nitrogen atoms in the TPPAPZ structure, TPPA and 2Ph-PZ were chosen as the model molecules instead of directly calculating TPPA-4Br and PZ. Atomic dipole moment-corrected Hirshfeld (ADCH) charge analysis reveals the local electronic structure distribution characteristics of TPPAPZ (Fig. 1f). In the TPPA unit, the nitrogen atoms exhibit ADCH values of 0.044 *e*, while in TPPAPZ they become 0.045 *e* and 0.041 *e*, indicating that this segment largely retains its intrinsic electronic characteristics within the extended conjugated framework, with only slight electronic polarization and symmetry breaking. In the 2Ph-PZ unit, the nitrogen atoms show an ADCH value of 0.012 *e*, and in TPPAPZ they vary only slightly within the range of 0.011–0.012 *e*, suggesting that this segment experiences minimal changes in its electronic environment and maintains high electronic structural stability. Overall, the ADCH results indicate that different conjugated segments in TPPAPZ exhibit distinct degrees of electron delocalization, leading to an electronically anisotropic yet conjugation-continuous electronic structure. Such an electronic configuration enables TPPAPZ, as an electrode material, to simultaneously possess electronically active sites and a structurally stable conjugated framework. The molecular electrostatic potential (ESP) surfaces provide a direct visualization of this donor-strength regulation across all three systems (Fig. 1g). In the TPPA monomer, the ESP distribution is relatively balanced, with moderately negative potential regions (blue) delocalized over the central nitrogen atoms, consistent with its moderate electron-donating ability. In contrast, the 2Ph-PZ monomer exhibits strongly negative ESP concentrated on the phenazine nitrogen atoms and the central aromatic core, reflecting its pronounced electron-rich character and higher electron density. Within the TPPAPZ structure, the ESP surface exhibits a pronounced intramolecular electronic gradient. Strongly negative potential regions are clearly distributed around the 2Ph-PZ moiety, whereas the negative potential on the TPPA segment is notably weaker in comparison. This spatially differentiated ESP distribution reflects the distinct local electronic environments arising from the different donor strengths of the units. The frontier molecular orbital analysis (Fig. 1h) further confirms the effectiveness of this modulation. The bandgaps of the monomers are large (TPPA, 4.20 eV; 2Ph-PZ, 3.69 eV). In TPPAPZ the bandgap narrows significantly to 3.42 eV, accompanied by an upshift of the Highest Occupied Molecular Orbital (HOMO) to −4.01 eV and a moderate stabilization of the Lowest Unoccupied Molecular Orbital (LUMO) to −0.59 eV. The modulated frontier-orbital structure and narrowed bandgap are consistent with enhanced electronic delocalization across the conjugated backbone, providing a favorable electronic-structure basis for p-type redox processes and charge transport.^39,40^

To further evaluate the effect of donor-strength differentiation, idealized pTPPA and pPZ polymer models, representing homogeneous TPPA–TPPA and PZ–PZ donor environments, respectively, were constructed (Fig. S12). Compared with pTPPA (3.69 eV) and pPZ (3.85 eV), TPPAPZ exhibits a narrower calculated bandgap (3.42 eV), further supporting the role of differentiated donor strengths in modulating the electronic structure of the all-donor conjugated backbone.

Overall, these computational results support the role of donor-strength differentiation in modulating the local electronic environment and frontier electronic structure of the all-donor conjugated backbone.^41^ By avoiding n-type acceptors, TPPAPZ retains the high-potential advantage of p-type polymers while achieving a high theoretical capacity.

### 2.2. Electrochemical performance in sodium half-cells

**Fig. 2:**
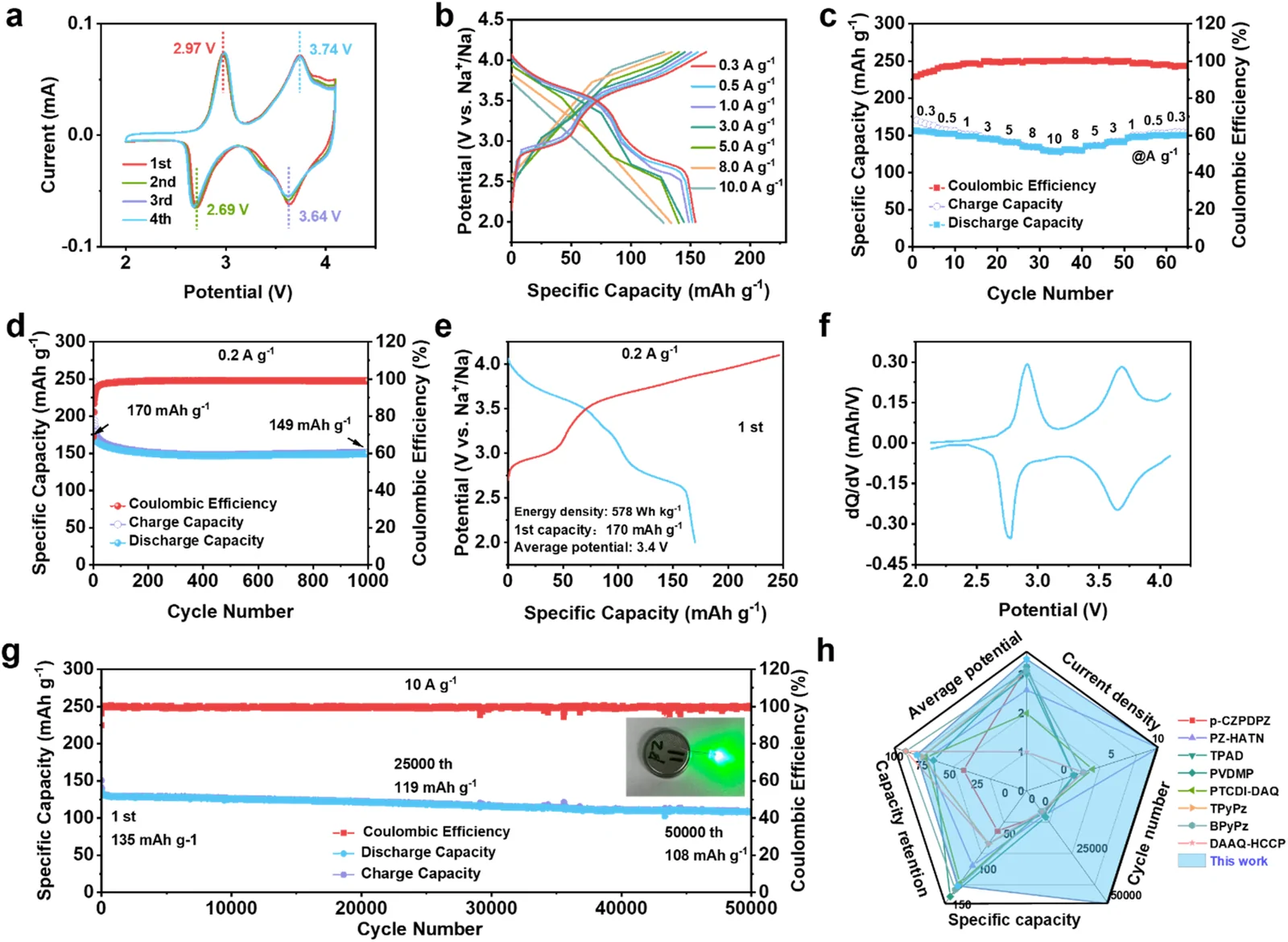
(a) Cyclic voltammograms at 0.5 mV s^−1^. (b) Charge–discharge profiles at various current densities. (c) Rate capability. (d) Long-term cycling performance at 0.2 A g^−1^. (e) Initial charge–discharge profile with energy density calculation. (f) Differential capacity (d*Q*/d*V*) plots during extended cycling. (g) Ultra-long cycling performance at 10 A g^−1^. (h) Comparison with representative state-of-the-art organic polymeric sodium-ion cathodes reported in the literature.

The electrochemical performance of TPPAPZ was systematically assessed in Na half-cells. Cyclic voltammetry (CV) curves revealed two pairs of well-defined redox peaks at 2.97/2.69 V and 3.74/3.64 V (Fig. 2a), indicative of two sequential and distinct redox processes upon charge–discharge. The narrow peak separations (0.28 V and 0.10 V) and excellent overlap of successive CV curves indicate strong redox reversibility. Galvanostatic charge–discharge profiles exhibited two clear voltage plateaus (Fig. 2b), aligning well with the CV redox peaks. The high symmetry between charge and discharge curves further supports the reversibility of the electrode reactions. Rate capability revealed robust high-rate behavior (Fig. 2c). At 0.3 A g^−1^, TPPAPZ delivered a discharge capacity of 160 mAh g^−1^. Even at an ultrahigh current density of 10 A g^−1^ (approximately 50C), it retained 128 mAh g^−1^, corresponding to a modest capacity retention of 80%. Upon returning to low current densities, the capacity fully recovered to its initial value. The excellent rate performance indicates favorable charge-transport and reaction kinetics in the TPPAPZ electrode. Additional evidence of superior kinetics is provided in the SI through CV curves at various scan rates and log(*i*_p_) *versus* log(*v*) plots for b-value calculations (Fig. S13 and S14). The ion diffusion coefficient, determined by galvanostatic intermittent titration technique (GITT), ranged from 10^−8^ to 10^−10^ cm^2^ s^−1^ (Fig. S15), significantly higher than those typically reported for organic polymer electrodes (around 10^−12^ cm^2^ s^−1^).^18^ This result supports rapid ion transport and is consistent with the excellent rate capability of the TPPAPZ electrode.

Long-term cycling stability was evaluated at 0.2 A g^−1^. The initial cycle delivered a high discharge capacity of 170 mAh g^−1^, which is slightly lower than the theoretical capacity (201 mAh g^−1^), likely because of incomplete utilization of redox-active sites and limitations in electronic/ionic transport within the polymer electrode.^42^ The calculated average potential of TPPAPZ was 3.4 V, corresponding to a remarkable energy density of 578 Wh kg^−1^ (based on the cathode active material). After 1000 cycles, the capacity remained at 149 mAh g^−1^, corresponding to 88% retention (Fig. 2d and e). In the initial charge–discharge cycle, the charge capacity exceeds the discharge capacity, resulting in a relatively low coulombic efficiency. This initial irreversibility may be associated with electrochemical activation of the pristine electrode, including partially irreversible PF_6_^−^ doping and interfacial processes during the first high-voltage oxidation.^43–45^ Moreover, the voltage plateaus remain well-defined even after 1000 cycles, indicating stable structural and redox behavior of the TPPAPZ electrode during prolonged cycling (Fig. S16). Differential capacity plots also show stable redox features throughout cycling (Fig. 2f). Cycling test at 10 A g^−1^ was further conducted to examine behavior under high-rate conditions. TPPAPZ completed full charge and discharge within one minute, delivering an initial capacity of 135 mAh g^−1^. After 25 000 cycles, it retained 119 mAh g^−1^ (88% retention), and even after 50 000 cycles, 108 mAh g^−1^ was maintained (80% retention). Post-cycling cells were still able to power an LED lamp, indicating continued cell functionality after extended cycling (Fig. 2g). Overall, extended conjugation in TPPAPZ substantially promotes charge redistribution and transport, thereby enhancing reaction kinetics and rate capability. At the same time, the deliberate exclusion of n-type redox-active functional groups enables the coexistence of high specific capacity and elevated discharge voltage. Consequently, the electrochemical performance of the TPPAPZ electrode outperforms most reported cathode materials for sodium-ion batteries (Fig. 2h, Table S1).

**Fig. 3:**
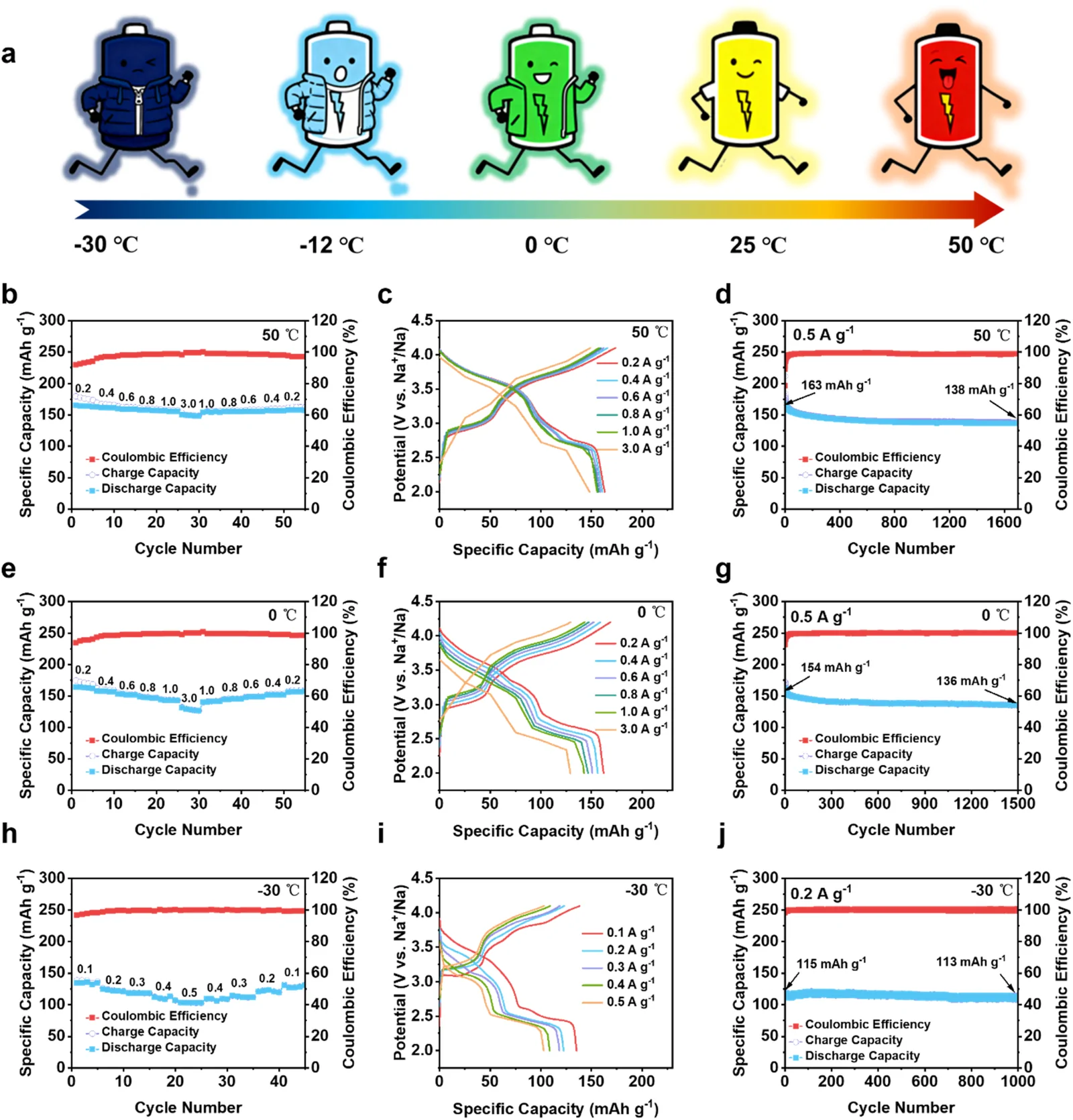
(a) Schematic illustration of battery operation across a wide temperature range. Electrochemical performance of TPPAPZ at 50 °C: (b) rate capability; (c) charge–discharge profiles under different current densities; (d) long-term cycling performance at 0.5 A g^−1^. Electrochemical performance of TPPAPZ at 0 °C: (e) rate capability; (f) charge–discharge profiles under different current densities; (g) long-term cycling performance at 0.5 A g^−1^. Electrochemical performance of TPPAPZ at −30 °C: (h) rate capability; (i) charge–discharge profiles under different current densities; (j) long-term cycling performance at 0.5 A g^−1^.

Electrochemical performance of TPPAPZ under extreme conditions was further evaluated in sodium dual-ion half-cells over a broad temperature range from −30 °C to 50 °C (Fig. 3a). At 50 °C, the electrode delivered 163 mAh g^−1^ at 0.2 A g^−1^, and even as the current density rose to 3 A g^−1^, capacity retention remained excellent at 150 mAh g^−1^, close to room-temperature behavior (Fig. 3b). Moreover, charge–discharge profiles across various rates and during prolonged cycling consistently showed two distinct plateaus with good reproducibility (Fig. 3c), indicating stable structural and electrochemical behavior under elevated temperatures. In long-term tests at 0.5 A g^−1^, an initial capacity of 163 mAh g^−1^ was obtained (Fig. S17), and after 1800 cycles, 138 mAh g^−1^ was retained (85% retention) with no signs of cell failure (Fig. 3d). This demonstrates that the TPPAPZ electrode maintains stable charge–discharge operation at elevated temperatures.

When the temperature was lowered to 0 °C, rate performance remained stable, yielding 163 mAh g^−1^ at 0.2 A g^−1^ (Fig. 3e). Although increasing the current density to 3 A g^−1^ slightly reduced the capacity to 129 mAh g^−1^ due to slower ion transport kinetics, the capacity fully recovered upon returning to lower rates, while the voltage profiles retained clear plateaus and high symmetry (Fig. 3f). Similarly, long-term cycling at 0.5 A g^−1^ started at 154 mAh g^−1^ (Fig. S18) and stabilized at 136 mAh g^−1^ after 1500 cycles, achieving 88% retention (Fig. 3g). Further cooling to −12 °C showed similar stability. After 500 cycles under similar conditions, the electrode maintained 133 mAh g^−1^ with 91% retention (Fig. S19 and S20). This demonstrates that TPPAPZ maintains stable and reversible electrochemical operation under sub-zero conditions.

At the extreme low temperature of −30 °C, the charge–discharge profiles exhibited increased polarization resulting from slower ion diffusion and reaction kinetics. At 0.1 A g^−1^, TPPAPZ delivered over 130 mAh g^−1^, and raising the current to 0.5 A g^−1^ maintained stable cycling at 100 mAh g^−1^, with complete recovery to 130 mAh g^−1^ upon returning to the lower rate (Fig. 3h). And, distinct plateaus of charge–discharge curves were still present (Fig. 3i). Furthermore, extended cycling at 0.2 A g^−1^ provided an initial 115 mAh g^−1^ (Fig. S21), which showed negligible decay to 113 mAh g^−1^ after 1000 cycles (Fig. 3j), while at 0.5 A g^−1^ over 3000 cycles, a stable 89 mAh g^−1^ was maintained (Fig. S22 and S23), indicating sustained electrochemical activity under extremely cold conditions.

The wide-temperature electrochemical operability of TPPAPZ may be associated with its regulated electronic structure and extended π-conjugated framework.^46^ The reduced band gap and enhanced electronic delocalization are expected to facilitate charge transport and help maintain electrochemical activity under low-temperature conditions, where ion transport and interfacial processes are intrinsically slowed. Overall, the electrochemical performance over the −30 to 50 °C range demonstrates the robust wide-temperature operability of TPPAPZ and provides useful molecular-design insights for organic cathodes in sodium dual-ion batteries.

### 2.3. Redox mechanism

**Fig. 4:**
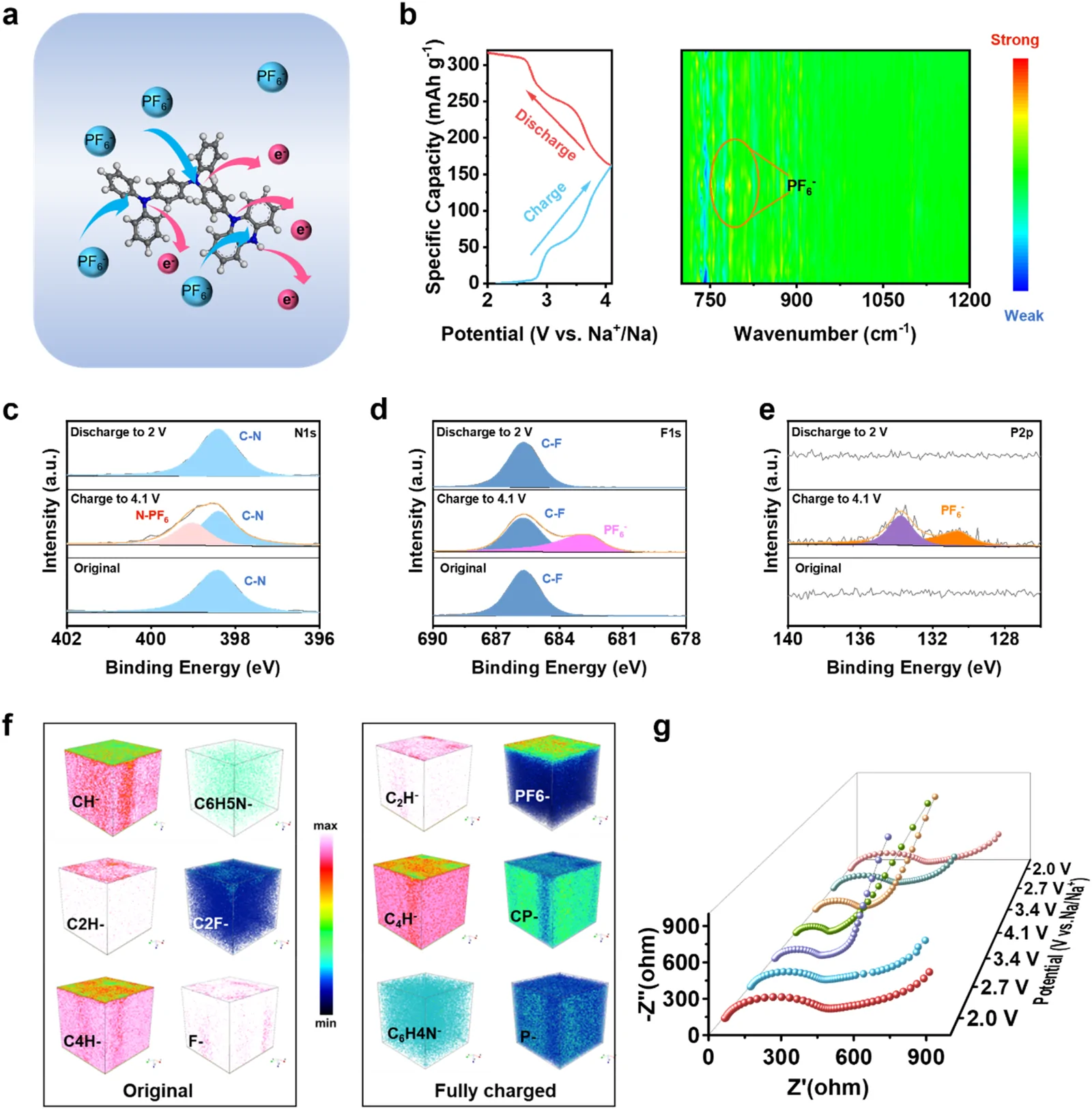
(a) Schematic diagram of ion transfer during the charging process of TPPAPZ. (b) *In situ* FTIR spectra. (c) *Ex situ* N 1s XPS spectra. (d) *Ex situ* F 1s XPS spectra. (e) *Ex situ* P 2p XPS spectra. (f) 3D ions concentration distributions and TOF-SIMS chemical mappings of the electrode. (g) EIS results for the TPPAPZ cathode during the charge and discharge processes.

As a fully p-type material, TPPAPZ is charged first during electrochemical cycling. During charging, oxidation takes place, in which the polymer loses electrons and is charge-compensated by the incorporation of PF_6_^−^ (Fig. 4a). The discharge process proceeds in the reverse manner. The ion storage mechanism of TPPAPZ as a sodium-ion battery cathode was investigated using *in situ* and *ex situ* spectroscopy. *In situ* Fourier-transform infrared (FTIR) spectroscopy was conducted using an electrochemical cell, with the spectrum of an uncycled TPPAPZ electrode having been subtracted as background. As shown in Fig. 4b, the PF_6_^−^-related signal at approximately 850 cm^−1^ progressively intensifies during charging and weakens upon discharge, consistent with the accumulation and subsequent removal of PF_6_^−^-related species during the p-type charge-compensation process. Complementary *ex situ* FTIR spectra exhibit a similar reversible evolution of the PF_6_^−^-related signal (Fig. S24). *Ex situ* X-ray photoelectron spectroscopy (XPS) was performed on the TPPAPZ electrode in its pristine, fully charged, and fully discharged states. The N 1s spectra (Fig. 4c) change upon charging, accompanied by the appearance of an additional component assigned to N-PF_6_^−^ interaction, which largely disappears after discharge. Meanwhile, the F 1s and P 2p signals increase upon charging and decrease upon subsequent discharge (Fig. 4d and e). Although the F 1s and P 2p signals may contain contributions from residual electrolyte or interphase species, their reversible evolution, together with the changes in N 1s and the FTIR results, supports reversible N-PF_6_^−^ interaction during the p-type charge-compensation process. XPS C 1s spectra and survey spectra are provided in the SI (Fig. S25–S30).

To gain insight into the distribution and behavior of anions within the cathode during charging, time-of-flight secondary ion mass spectrometry (TOF-SIMS) was employed to characterize the electrodes in pristine and charged states. Fig. S31 shows 2D ion distribution maps of the charged electrode. The charged electrode exhibits prominent signals of PF_6_^−^, P^−^ and CP^−^, with a color scale from yellow (high intensity) to black (low intensity), revealing a clearly non-uniform distribution across the electrode surface. PF_6_^−^ appears as distinct patchy high-concentration regions, indicating that PF_6_^−^ from the electrolyte is incorporated into the polymer matrix *via* an anion-doping mechanism during charging, compensating the positive charge at oxidized nitrogen active sites. P^−^ and CP^−^ follow a broadly similar but more diffuse pattern, consistent with partial decomposition or fragmentation of PF_6_^−^. Fig. 4f provides 3D reconstructions of these ions. In the initial state, the electrode only shows ion fragments of carbon skeletons, such as CH^−^, C_2_H^−^, and C_6_H_5_N^−^, along with C_2_F^−^ fragments derived from the PVDF binder. These fragments are uniformly distributed across the electrode. In contrast, after full charging, ion fragments corresponding to the carbon skeleton of TPPAPZ appear, along with signals of PF_6_^−^, CP^−^, and P^−^. The CP^−^ and P^−^ fragments originate from the ionization and decomposition of the PF_6_^−^ anion. This indicates that upon charging, TPPAPZ undergoes an oxidation reaction and loses electrons to form a positively charged species, which then facilitates the incorporation of PF_6_^−^ into the electrode to maintain charge neutrality. Notably, PF_6_^−^ shows significant surface enrichment, whereas CP^−^ and P^−^ remain uniformly distributed within the electrode. This observation suggests that the surface enrichment of PF_6_^−^ is primarily due to residual electrolyte. These results provide spatially resolved evidence for the increased presence of PF_6_^−^ in the charged TPPAPZ electrode, consistent with anion involvement during the charging process. They also reveal pronounced heterogeneity and structural dependence of anion transport and trapping within the polymer electrode.

Furthermore, electrochemical impedance spectroscopy (EIS) provided additional information (Fig. 4g). During charging (PF_6_^−^ insertion), the high-frequency semicircle and mid-to-low-frequency Warburg tail decrease in size, resulting in a lower overall impedance. The opposite trend occurs upon discharge (PF_6_^−^ extraction). This impedance evolution is consistent with p-type doping behavior, where oxidation generates cation radicals or polarons at nitrogen sites, increasing carrier concentration and electronic conductivity. De-doping reverses these effects, leading to higher charge-transfer and diffusion resistances. Thus, the strong correlation between impedance and state-of-charge (SOC) supports the reversible PF_6_^−^ intercalation/deintercalation mechanism. Scanning electron microscopy (SEM) imaging of TPPAPZ at different charge states (Fig. S32) revealed that the spherical morphology was preserved throughout cycling, with no evidence of structural collapse, enabling structural stability and reversibility. Elemental mapping indirectly also supports PF_6_^−^ involvement, where F and P signals are prominent on the surface in the fully charged state but absent in pristine and discharged states. These observations are consistent with anion insertion and removal. Taken together, the corresponding charging and discharging mechanism is summarized in Fig. S33.

**Fig. 5:**
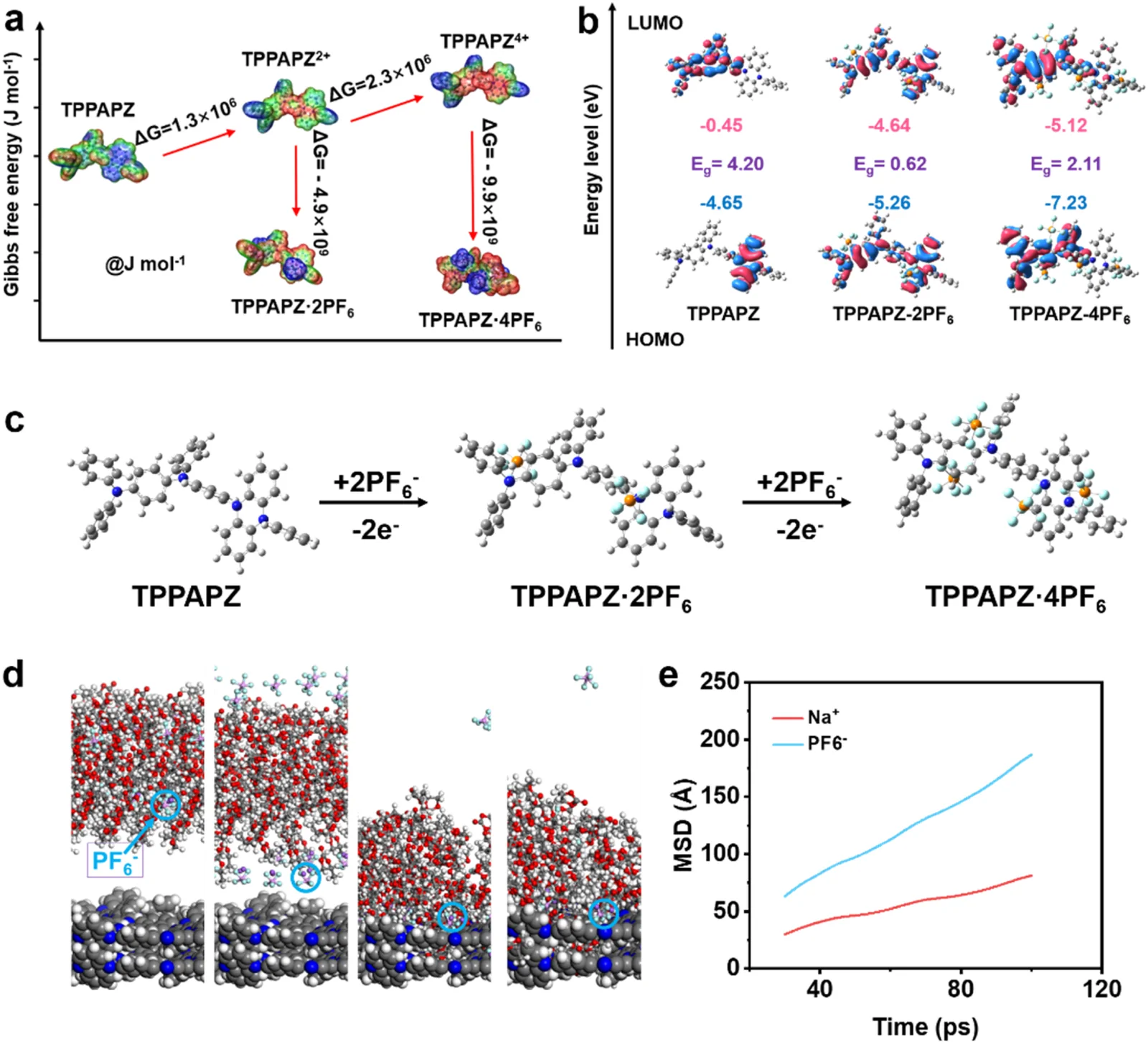
(a) DFT-calculated molecular electrostatic potentials and total Gibbs free energy changes. (b) HOMO–LUMO energy levels and bandgap values. (c) Schematic illustration of the charging process. (d) The adsorption process of PF_6_^−^ by the TPPAPZ electrode as simulated by molecular dynamics. (e) The mean square displacement (MSD) curves for Na^+^ and PF_6_^−^.

The Gibbs free energy of TPPAPZ during the oxidation process was calculated using density functional theory (DFT). Gibbs free energy changes are positive from TPPAPZ to TPPAPZ^2+^ and TPPAPZ^4+^ (Fig. 5a), suggesting that the oxidation steps are non-spontaneous and require external applied voltage. In contrast, anion coordination to form TPPAPZ-2PF_6_ and TPPAPZ-4PF_6_ is spontaneous (Δ*G* < 0). Fig. 5b shows the frontier molecular orbitals. Upon two-electron oxidation (TPPAPZ-2PF_6_), the HOMO drops to −5.26 eV, the LUMO drops to −4.64 eV, and the bandgap decreases to 0.62 eV, reflecting enhanced intramolecular charge delocalization. Full four-electron oxidation (TPPAPZ-4PF_6_) lowers both orbitals (HOMO: −7.23 eV; LUMO: −5.12 eV), yielding a bandgap of 2.11 eV. The lowered energy level value is consistent with electrostatic stabilization by counter anions. The smallest bandgap appears in the di-cationic state, associated with planarization of the TPPAPZ^2+^, which increases π-electron delocalization. Further oxidation introduces electrostatic repulsion and structural distortion, which disrupt conjugation and widen the gap despite additional anion stabilization. The two-electron oxidized state therefore corresponds to the most favorable structure for conjugation and charge delocalization in this polymer. Guided by the CV profiles, DFT was employed to simulate the two-step redox process (Fig. 5c). In the first step, TPPAPZ loses two electrons, with oxidation occurring at one nitrogen site in each of the TPPA and PZ units, followed by spontaneous coordination with two PF_6_^−^ anions. The second step involves further oxidation of the remaining nitrogen atoms and binding of two additional PF_6_^−^. The discharge process is the opposite.

Molecular dynamics (MD) simulations were performed to investigate ion transport between the electrolyte and electrode during charging (Fig. S34). The results reveal that, upon oxidation of the nitrogen active sites, PF_6_^−^ gradually migrate toward the cathode and eventually adsorb tightly onto these sites (Fig. 5d). Fig. 5e presents the mean square displacement (MSD) curves for Na^+^ and PF_6_^−^ ions in the oxidized TPPAPZ system as a function of time. Within the 30−100 ps timeframe, the MSD of PF_6_^−^ exceeds that of Na^+^, resulting in an enhanced diffusion coefficient for the anion. This disparity suggests that PF_6_^−^ exhibits higher apparent mobility than Na^+^ in the simulated oxidized TPPAPZ system. Such differential ion diffusion can be explained by the charge compensation mechanism in p-type redox-active polymer cathodes. During oxidation (charging), the nitrogen sites on the polymer backbone become positively charged (N^+^), attracting PF_6_^−^*via* strong electrostatic interactions. These anions are preferentially drawn toward the oxidized polymer phase to maintain electroneutrality and show faster apparent transport under the simulated charging conditions. In contrast, Na^+^ ions, primarily confined to the electrolyte phase or the polymer–electrolyte interface, experience stronger solvation and steric hindrance, resulting in restricted mobility. These findings align closely with radial distribution function (RDF) analysis (Fig. S35). The total RDF displays a sharp, intense peak at approximately 3.5–4.0 Å, characteristic of a highly ordered first coordination shell. This feature is dominated by the PF_6_^−^ RDF, which exhibits a prominent peak in the same range, reflecting strong electrostatic attraction and close coordination between PF_6_^−^ and the positively charged nitrogen sites. In contrast, the Na^+^ RDF (red curve) exhibits only a weaker, broader peak within 5 Å suggesting weaker direct coordination dominated by solvation effects. At longer distances (>8 Å), all curves approach unity, consistent with long-range disorder. This RDF analysis supports the presence of an anion-dominated doping mechanism in the oxidized state. PF_6_^−^ preferentially associates with the positive centers for charge compensation, whereas Na^+^ coordination remains relatively loose. Together with the MSD results, these observations support an anion-dominated charge-compensation process, which is likely associated with the high-rate charge–discharge behavior observed experimentally.

### 2.4. Electrochemical performance in sodium full-cells

**Fig. 6:**
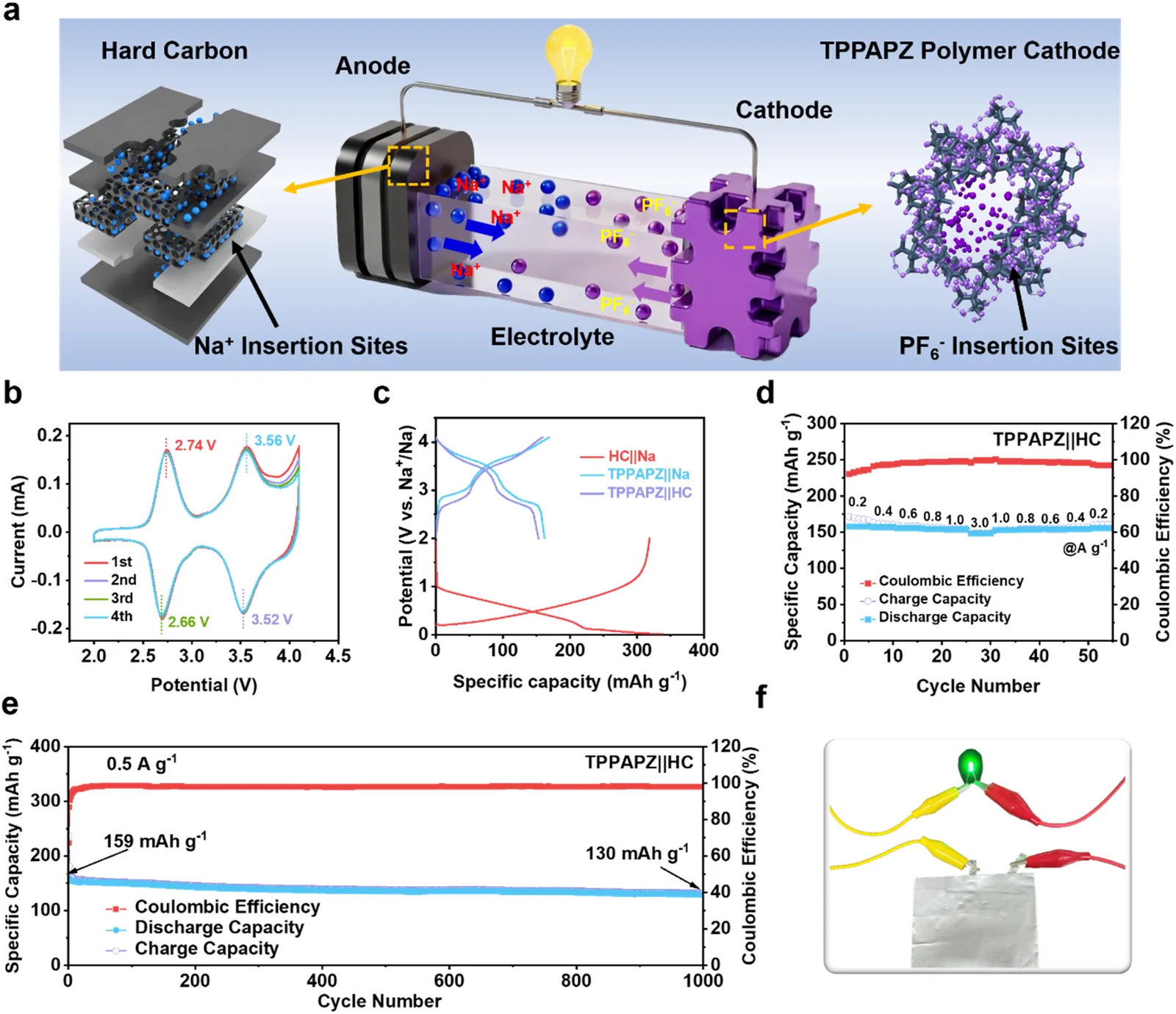
(a) Schematic illustration of the TPPAPZ‖HC full sodium-ion battery. (b) Cyclic voltammograms at 0.5 mV s^−1^. (c) Charge–discharge profiles of HC‖Na half-cell, TPPAPZ‖Na half-cell, and TPPAPZ‖HC full-cell. (d) Rate capability of the TPPAPZ‖HC full-cell from 0.2 to 3 A g^−1^ (cathode basis). (e) Long-term cycling performance of the TPPAPZ‖HC full-cell at 0.5 A g^−1^. (f) Demonstration of a pouch cell powering an LED.

The electrochemical performance of TPPAPZ was further evaluated in practical full-cell configurations. Full sodium dual-ion batteries were assembled using hard carbon (HC) as the anode and TPPAPZ polymer as the cathode (Fig. 6a). The cathode mass loading and electrolyte conditions are provided in the SI to clarify the practical testing parameters. The cyclic voltammetry (CV) curves of the TPPAPZ‖HC full cell align closely with those of the TPPAPZ half-cell, showing a two-step redox process (Fig. 6b). However, the redox peaks shifted downward by approximately 0.1 V overall, attributable to the redox potential of the HC anode. This observation was corroborated by the galvanostatic charge–discharge profiles (Fig. 6c). Multiple CV scans overlapped almost perfectly, indicating high redox reversibility of TPPAPZ in the full-cell setup. At various scan rates, all curves displayed broad yet symmetric redox peaks (oxidation at ∼2.71 V and 3.53 V, reduction at ∼2.65 V and 3.45 V), indicating highly reversible behavior. Even at the highest scan rate, peak symmetry is maintained with minimal position shift and negligible separation widening, underscoring excellent electrochemical reversibility and fast redox kinetics (Fig. S36). Rate capability tests further illustrate the stability of the TPPAPZ‖HC cell (Fig. 6d). At 0.2 A g^−1^, it delivered 158 mAh g^−1^ with an average voltage of 3.32 V. As the current density increased stepwise to 1 A g^−1^, capacity decline was negligible, forming an almost flat profile. Even at 3 A g^−1^, 148 mAh g^−1^ was retained (a minor loss of 10 mAh g^−1^). Upon returning to 0.2 A g^−1^, the capacity recovered fully to 156 mAh g^−1^ (Fig. S37). This rate behavior remains stable across the tested current range. Long-term cycling stability was also evaluated. At 0.5 A g^−1^, the cell provided an initial discharge capacity of 159 mAh g^−1^. After 1000 cycles, 130 mAh g^−1^ remained, which corresponds to 82% retention (Fig. 6e). After cycling, the cells remained capable of powering a commercial LED lamp, verifying retained practical functionality. When the current density was raised to 1 A g^−1^, the capacity stabilized at 119 mAh g^−1^ after 2000 cycles (Fig. S38). A laboratory-scale pouch cell was also assembled for preliminary demonstration. After charging, the pouch cell powered an LED lamp under normal operating conditions, indicating device-level feasibility (Fig. 6f). These full-cell and pouch-cell results further demonstrate the feasibility of TPPAPZ for sodium dual-ion battery applications.

## 3 Conclusions

This study proposes a donor-strength-tuning molecular design strategy for a fully p-type organic cathode, exemplified by TPPAPZ, which addresses the inherent voltage-capacity trade-off in conventional bipolar organic electrodes. By integrating two donor moieties with distinct electron-donating strengths, TPPAPZ achieves controlled electronic structure modulation and efficient charge transport. The material exhibits a high average discharge voltage of 3.4 V, exceptional high-rate capability (128 mAh g^−1^ at 10 A g^−1^), ultra-long cycling stability (80% retention after 50 000 cycles), and robust operation over a wide temperature window (−30 °C to 50 °C). Full-cell tests confirm stable cycling and competitive energy density, demonstrating the competitive electrochemical performance of TPPAPZ as an organic cathode for wide-temperature sodium dual-ion batteries.

Beyond the superior material performance, this work proposes a generalizable design strategy for high-voltage organic cathodes. The differentiated donor strengths in TPPAPZ enable modulation of the local electronic environment and frontier electronic structure while preserving the high-voltage characteristics of p-type redox chemistry. Together with the extended π-conjugated framework, these features contribute to its favorable electrochemical performance. It provides insights into structure–property relationships and offers a useful framework for future polymer electrode design toward sustainable and high-performance electrochemical energy storage.

## Author contributions

S. L. writing – original draft, conceptualization, data curation; P. L. funding acquisition, supervision, writing – review and editing; L. L, Y. Z. and X. Z. data curation, formal analysis; L. W., F. C. W., T. Z., and Q. L. resources, discussion. All authors discussed the results and contributed to manuscript revision.

## Conflicts of interest

There are no conflicts to declare.

## Supplementary Material

SC-OLF-D6SC05745D-s001

## Data Availability

All the data required for all the conclusions drawn in the paper (such as the characterization of materials, the long cycle performance of batteries, the rate performance, *etc.*) have been presented in the main body of the paper and the supplementary information (SI) section. The data that support the findings of this study are available from the corresponding author upon reasonable request. Supplementary information: experimental and computational details, supplementary characterization and electrochemical data, and supporting figures and tables. See DOI: https://doi.org/10.1039/d6sc05745d.
