# Supplementary material for: Donor-strength tuning enables a high-voltage all-p-type polymer cathode with fast redox kinetics for wide-temperature sodium dual-ion batteries
Source: Chem Sci. 2026 Aug 28;17(40):19495–505. doi: 10.1039/d6sc05745d (PMC13532545; doi:10.1039/d6sc05745d)
Supplement: Supplementary file 1 [file SC-017-D6SC05745D-s001.pdf]

Supplementary Information (SI) for Chemical Science.
This journal is © The Royal Society of Chemistry 2026

Supporting Information

Donor-Strength Tuning Enables a High-Voltage All-p-Type Polymer Cathode with Fast Redox Kinetics for Wide-Temperature Sodium Dual-Ion Batteries

Shuai Liu[a], Puiki Leung[, a], Linyang Li[a], Yong Zuo[a], Lei Wei*[, b], Frank C Walsh[c], Xun Zhu[a], Tianshou Zhao*[, b] and Qiang Liao[a]*

Materials characterizations

These are the characterization instruments used in this experiment. Fourier transform infrared (FTIR, Thermo Fisher Scientific Nicolet iS20, America), Nuclear magnetic resonance (NMR, Bruker Avance Neo, Germany), X-ray photoelectron spectrometer (XPS, Thermo Scientific K-Alpha, America). X-ray diffractometer (XRD, Rigaku SmartLab SE, Japan), Scanning electron microscopy (SEM, TESCAN MIRA LMS, Czech Republic). High-resolution mass spectrometry (HRMS, Bruker ESI-Q-TOF MS/MS, Germany), Transmission electron microscopy (TEM, JEOL JEM-F200, Japan), Liquid nuclear magnetic resonance spectroscopy (NMR, Bruker Avance NEO, Germany), High-temperature gel permeation chromatography (GPC, Waters 1525 pump with Waters 2414 refractive index detector).

Electrode preparation

The battery assembly materials, including the battery casing, electrolyte, sodium metal anode, etc., were all purchased from dodochem.com.

The electrode is prepared by mixing TPPAPZ, Ketjen Black, PVDF binder according to the ratio of 6:3:1 and adding NMP to prepare a uniform slurry. Apply the paste evenly to the aluminum foil collector with carbon layer. Vacuum 80 degrees after 12 hours cut into a diameter of 12 mm circular electrode. The load of electrode sheet is about 1 mg cm^{-2}. At the time of in situ testing, the mass ratio of TPPAPZ, Super p, PVDF on the electrode was 8:1:1.

Battery assembly

The battery model used in the lab is CR2032. In the half battery, the anode electrode uses sodium metal, the electrolyte is 1 M $NaPF_6$-PC with 5% FEC, the diaphragm is glass fiber membrane GF/D, and the cathode electrode uses TPPAPZ. After the electrode assembly was completed, the electrochemical performance was tested after standing for 8 hours.

The assembly of a full battery is similar to that of a half-cell, with the difference being that the anode electrode material is replaced with hard carbon. The N/P ratio was intentionally set in the range of 1.1–1.5 to ensure sufficient excess capacity on the anode side. This design minimizes the influence of the anode on the overall electrochemical

performance and allows for a more accurate evaluation of the intrinsic properties of the TPPAPZ cathode material.

The assembly of the pouch cell battery involves TPPAPZ, paired with a hard carbon anode. The electrolyte employed is 1 M $NaPF_6$-PC with 5% FEC. For the electrode and separator preparation, both the positive and negative electrodes are fabricated as rectangular sheets measuring 4 × 6 cm. The glass fiber separator, cut to dimensions of 4.3 × 6.3 cm, is slightly larger than the electrodes to prevent direct contact and potential short. The cell is assembled by sandwiching the separator between the cathode and anode, followed by encapsulation in an aluminum-plastic laminated film pouch.

Electrochemical characterizations

The cyclic voltammetry (CV) and electrochemical impedance spectroscopy (EIS) were performed on the DH7003 electrochemical workstation. Rate performances, galvanostatic charge-discharge (GCD), galvanostatic intermittent titration technique (GITT) tests, were conducted on the Neware MHW-100-2-160CH test system.

Density functional theory

Density functional theory (DFT) calculations were performed using Gaussian 09 software. The geometric configuration and frequency of organic molecules at different discharge states were calculated at the B3LYP/6-311G (d, p) level. The molecule energy is optimized by the B3LYP/6-311G (d, p) basis set.

The calculation of theoretical capacity and energy density

Theoretical capacity C (mAh g^{-1}) was calculated using the following equation:

$$C = \frac{1000nF}{3600(Mw)}$$

Where n and Mw is the number of charge carrier and the molecular weight of the active material, respectively, F is the Faraday constant (96485 C/mol). Therefore, the theoretical capacity is calculated using the equation of C=201 mAh g^{-1}.

Energy density (Wh kg^{-1}) was calculated using the following equation:

$$E = V \times C$$

Where V and C is the average discharge potential (V) and specific discharge capacity (Ah kg^{-1}), respectively. Or *E* can obtain it by integrating the discharge curve.

Materials and Methods

All reagents and solvents were purchased from Aladdin (Shanghai, China) and used as received without further purification.

Synthesis of N^1,N^1,N^4,N^4-tetrakis(4-bromophenyl)benzene-1,4-diamine (TPPA-4Br)[1, 2]

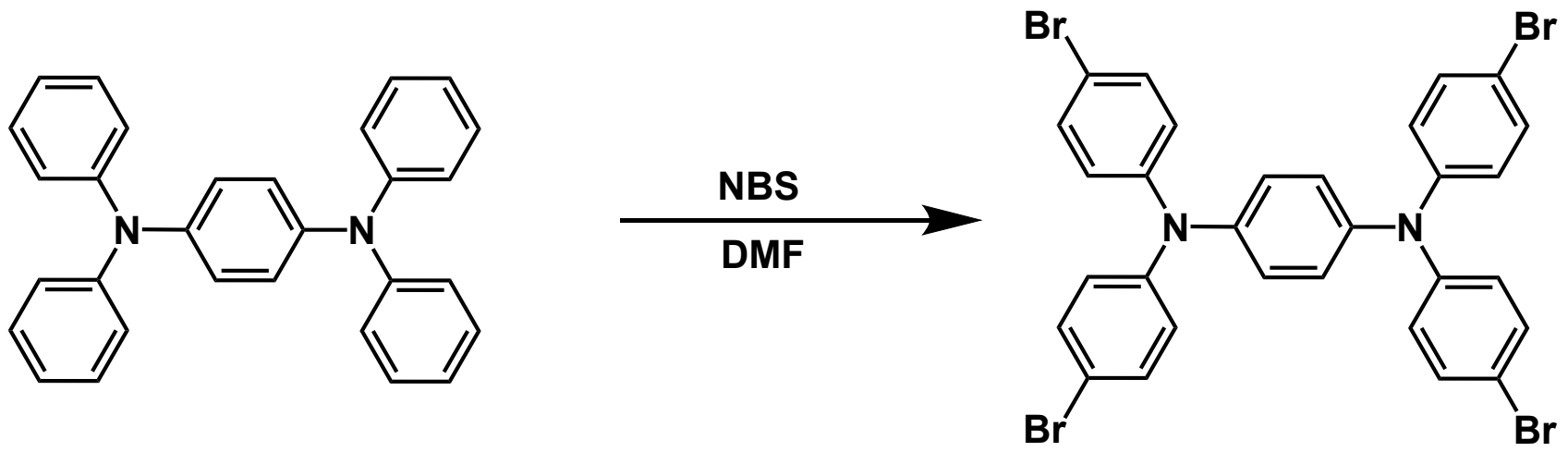

Figure S1. Synthesis scheme for TPPA-4Br.

The compound was synthesized according to the literature method. N^1,N^1,N^4,N^4-Tetraphenylbenzene-1,4-diamine (1.00 g, 2.42 mmol, 1 equiv) was suspended in 40 mL of DMF and cooled to 0 °C in an ice bath. N-Bromosuccinimide (1.94 g, 10.8 mmol, 4.5 equiv) was dissolved in 20 mL of DMF and added slowly to the above solution of N^1,N^1,N^4,N^4-tetraphenylbenzene-1,4-diamine over 30 min using a syringe pump. After the addition was complete, the reaction mixture was stirred at 0 °C for an additional 10 min and then allowed to warm to room temperature. The green suspension was stirred overnight, after which it was poured into water (500 mL). The resulting precipitate was collected by filtration and recrystallized from chloroform (200 mL). TPPA-4Br was isolated as a white powder by filtration, washed three times with chloroform, and dried under vacuum (1.39 g, 79% yield). ^{1}H-NMR (600 MHz, $CDCl_3$): δ (ppm) = 7.33 (d, 8H, H_1), 6.93 (m, 12H, $H_{2,3}$).[1]

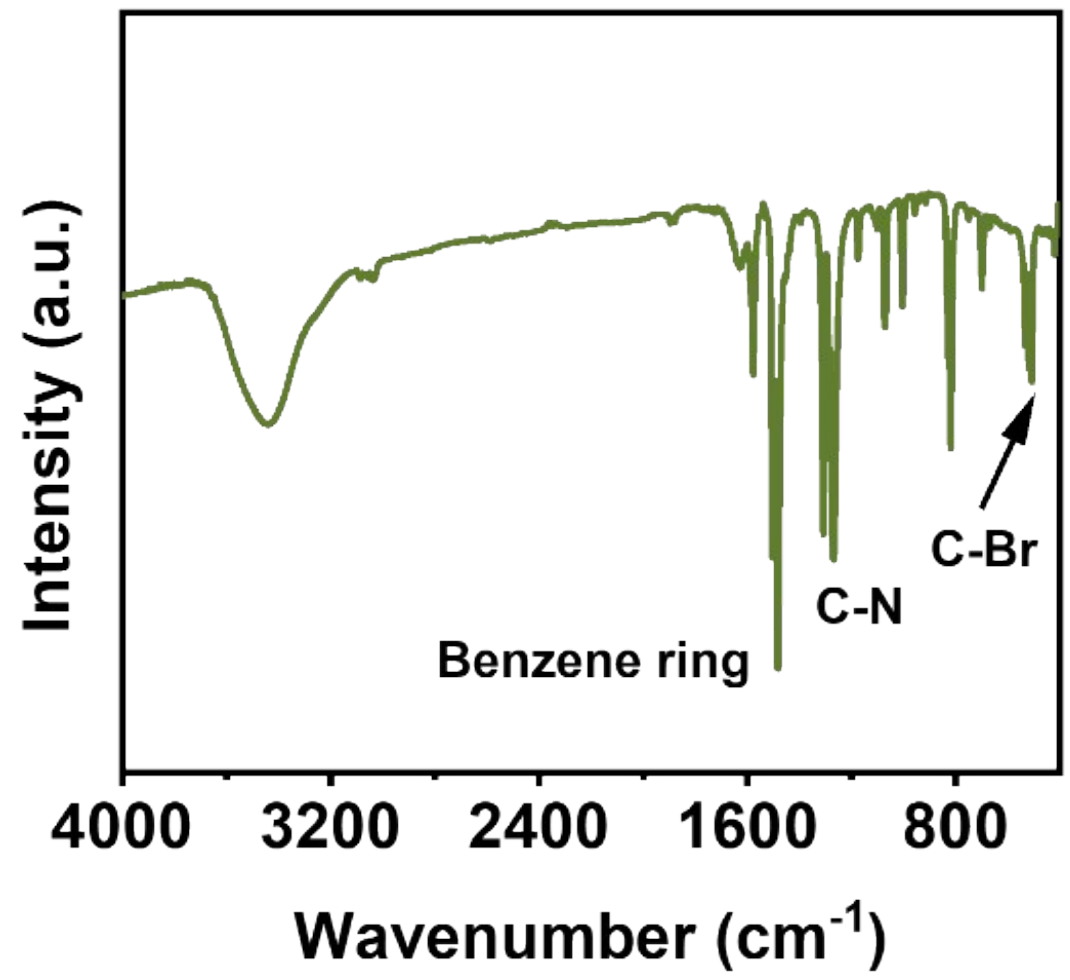

Figure S2. FTIR of TPPA-4Br.

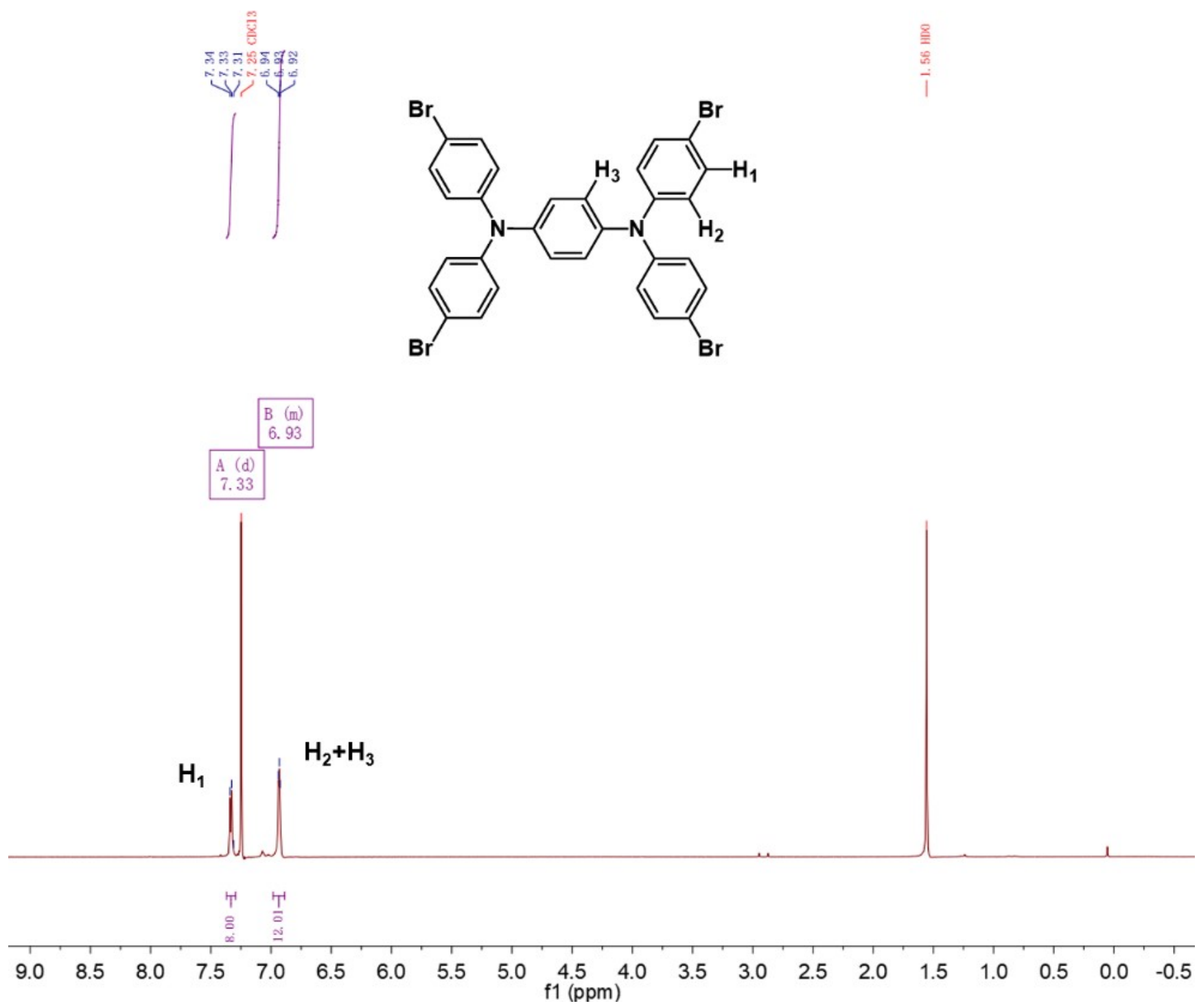

Figure S3. ^{1}H-NMR spectra of TPPA-4Br.

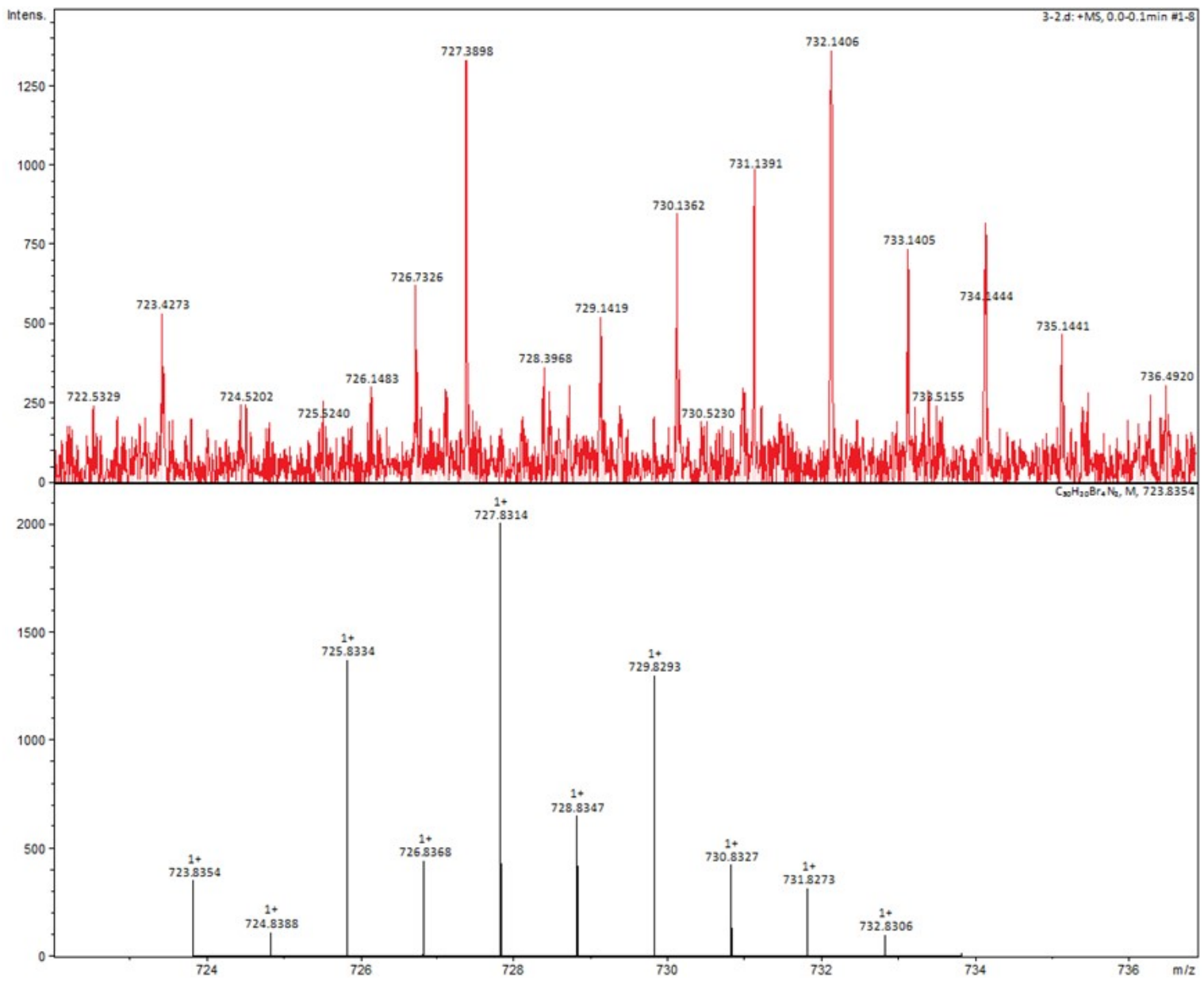

Figure S4. ESI-MS of TPPA-4Br (M=728).

Synthesis of TPPAPZ

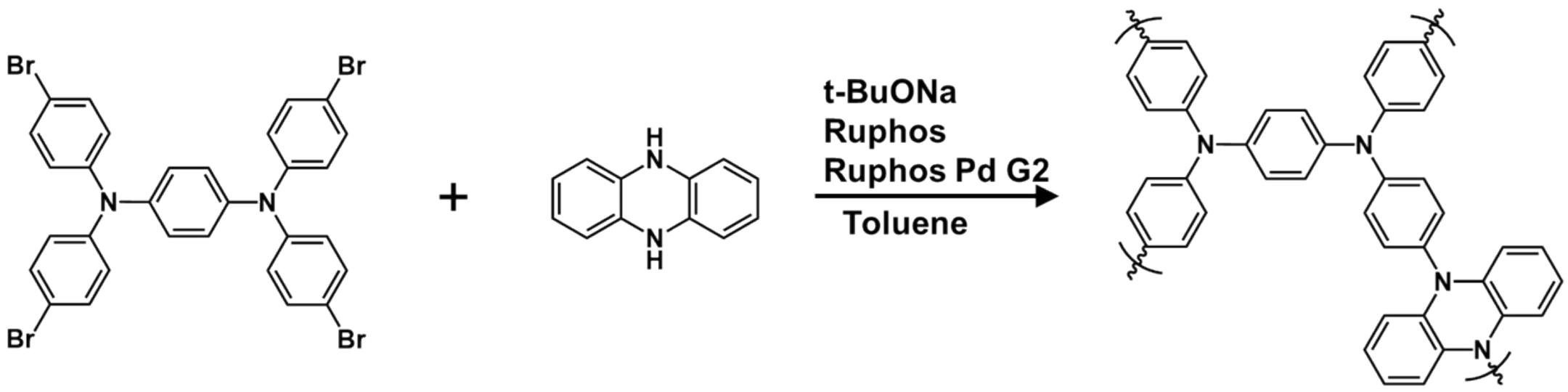

Figure S5. Synthesis scheme for TPPAPZ.

Under a nitrogen atmosphere, N^1,N^1,N^4,N^4-tetrakis(4-bromophenyl)benzene-1,4-diamine (TPPA-4Br, 364 mg, 0.5 mmol, 1 equiv), 5,10-dihydrophenazine (PZ, 183 mg, 1 mmol, 2 equiv), 2-Dicyclohexylphosphino-2′,6′-diisopropoxybiphenyl (RuPhos, 14 mg, 0.03 mmol, 0.06 equiv), RuPhos Pd G2 (23 mg, 0.03 mmol, 0.06 equiv), sodium tert-butoxide (481 mg, 5 mmol, 10 equiv), and toluene (30 mL) were added to a flask. The flask was sealed and the mixture was heated at 110 °C with stirring for 3 days. After completion of the reaction, the mixture was filtered to afford a gray solid powder, which was washed with petroleum ether, acetone, methanol, and deionized water. The crude product was purified by Soxhlet extraction with tetrahydrofuran and methanol for 48 hours, followed by drying under vacuum to yield a gray powder (349 mg, 91% yield).

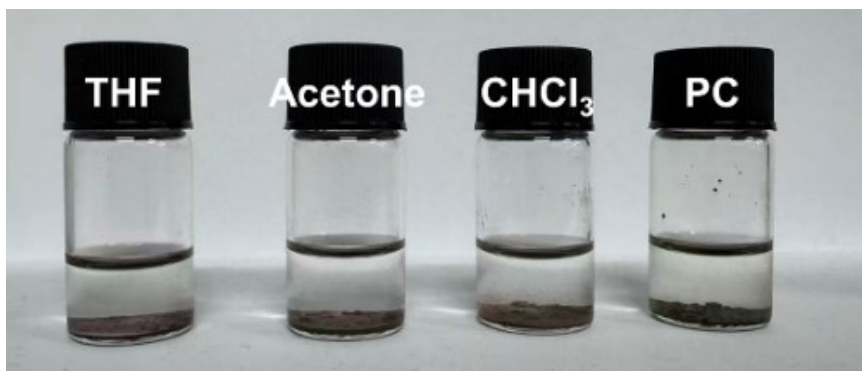

Figure S6. Optical photographs of TPPAPZ powder in common organic solvents.

Optical photographs of TPPAPZ powder added to Tetrahydrofuran (THF), acetone, $CHCl_3$, and propylene carbonate (PC), respectively. The suspensions were sonicated for 10 minutes and then allowed to stand overnight.

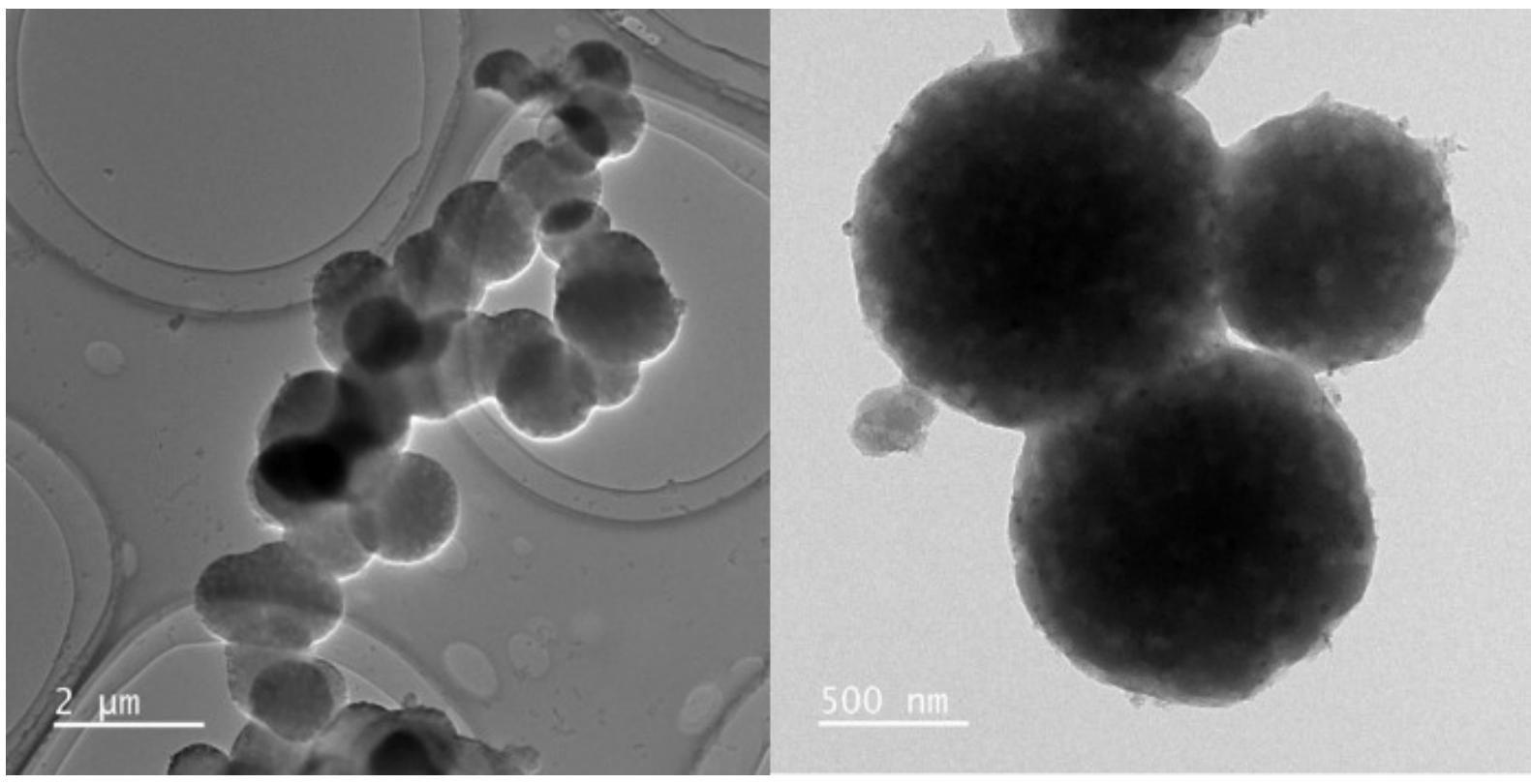

Figure S7. TEM images of TPPAPZ powder.

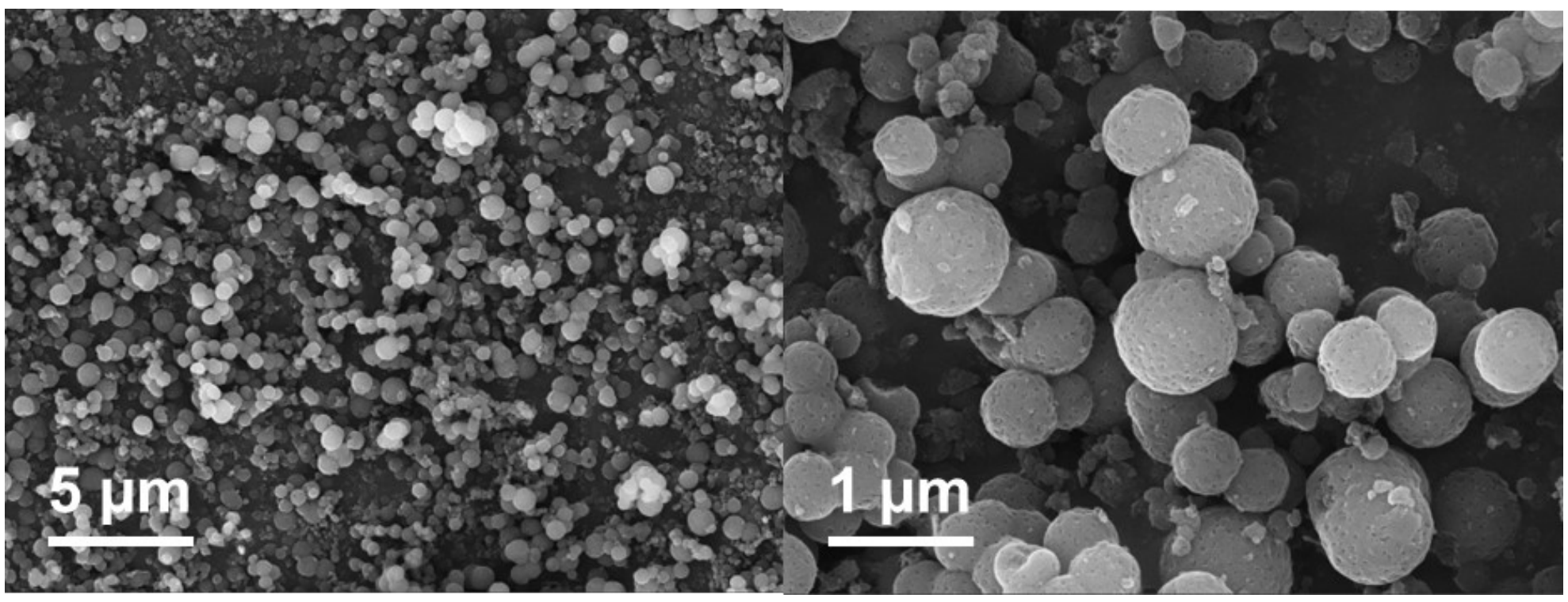

Figure S8. SEM images of TPPAPZ powder.

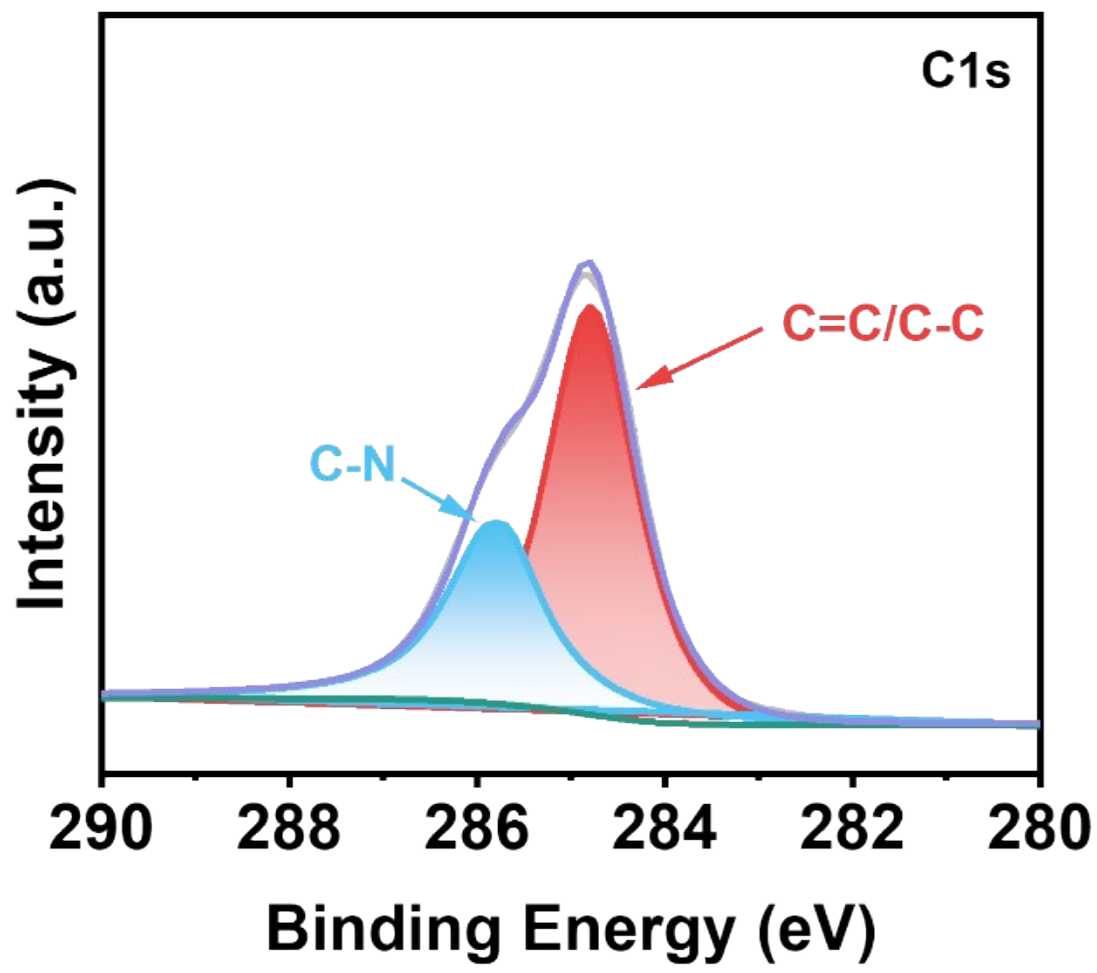

Figure S9. High-resolution C 1s XPS spectrum of TPPAPZ.

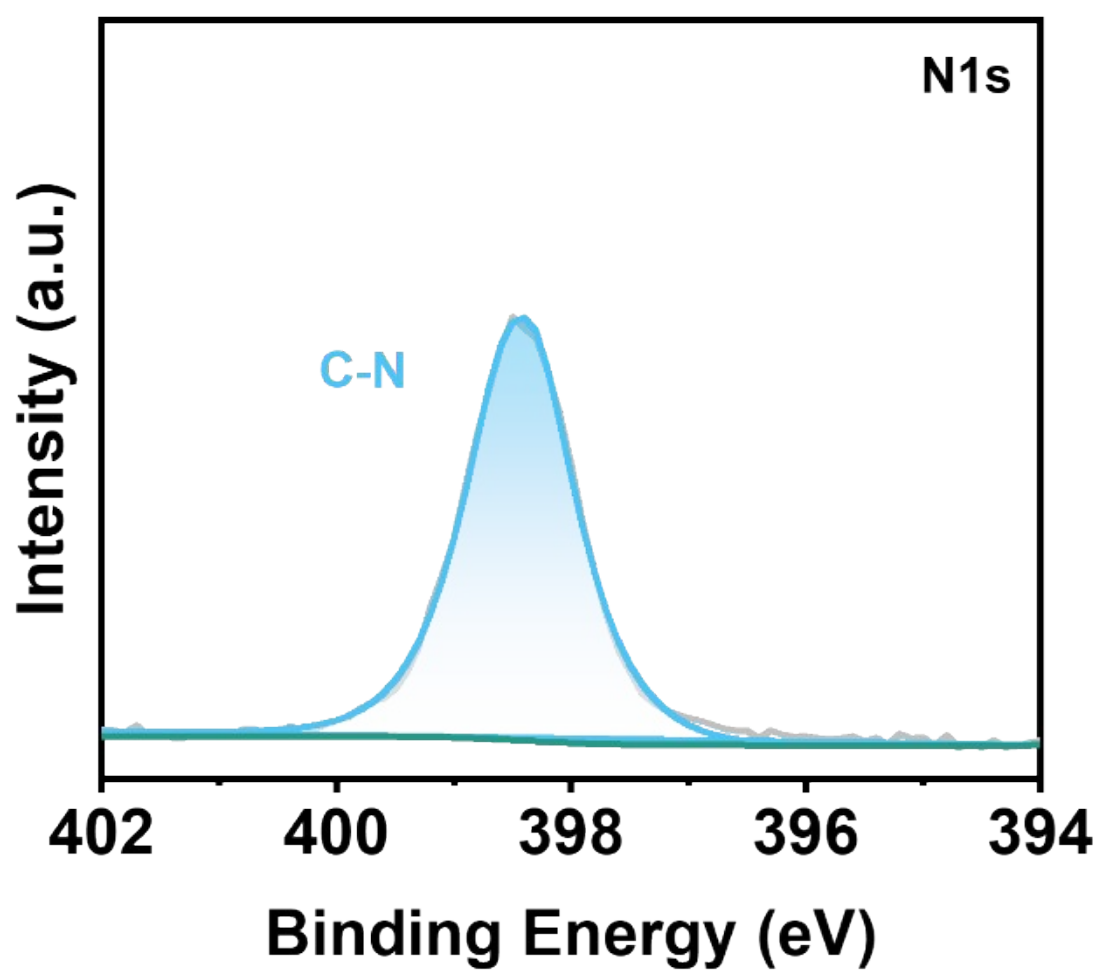

Figure S10. High-resolution N 1s XPS spectrum of TPPAPZ.

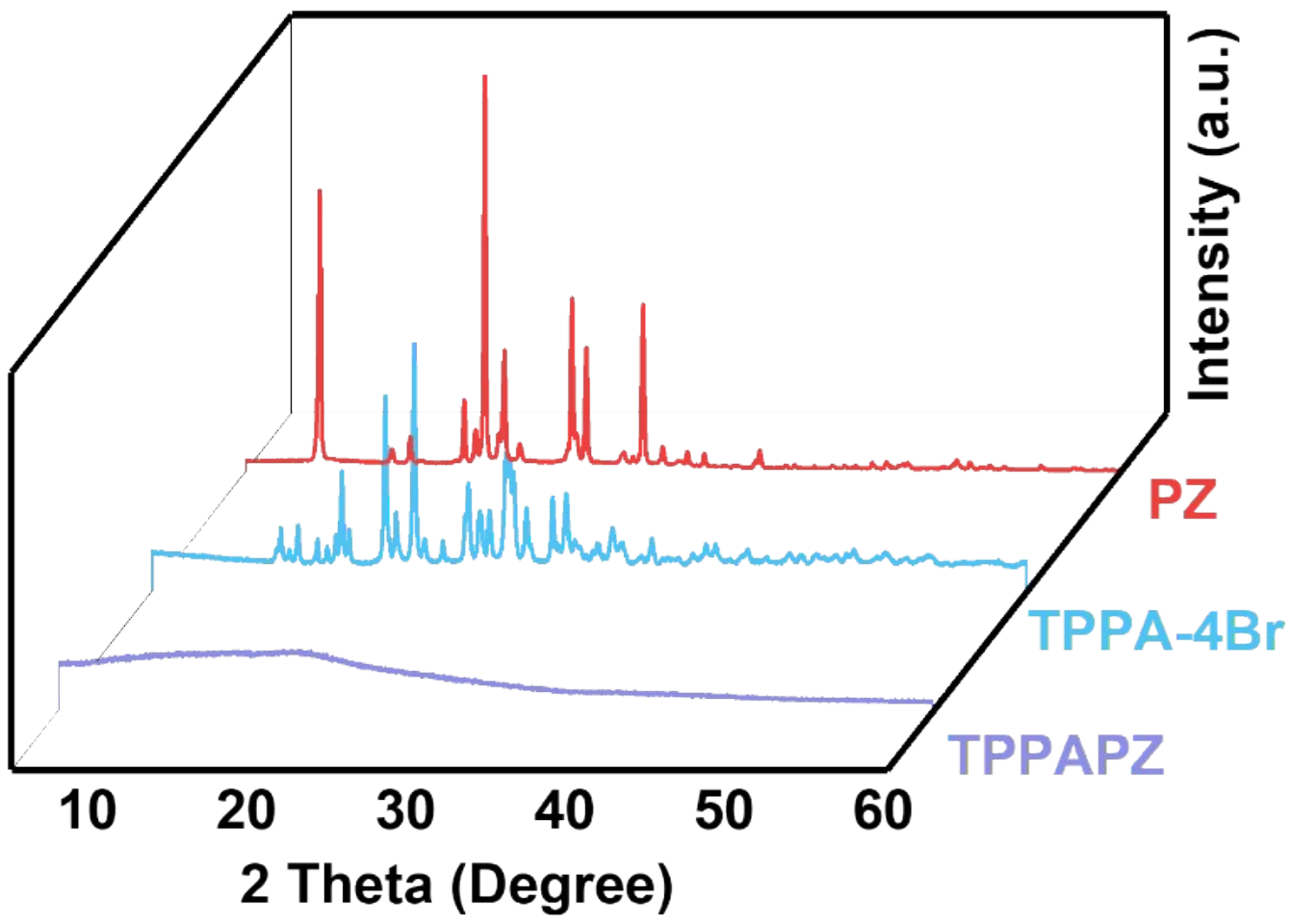

Figure S11. XRD patterns of TPPA-Br, PZ, and TPPAPZ.

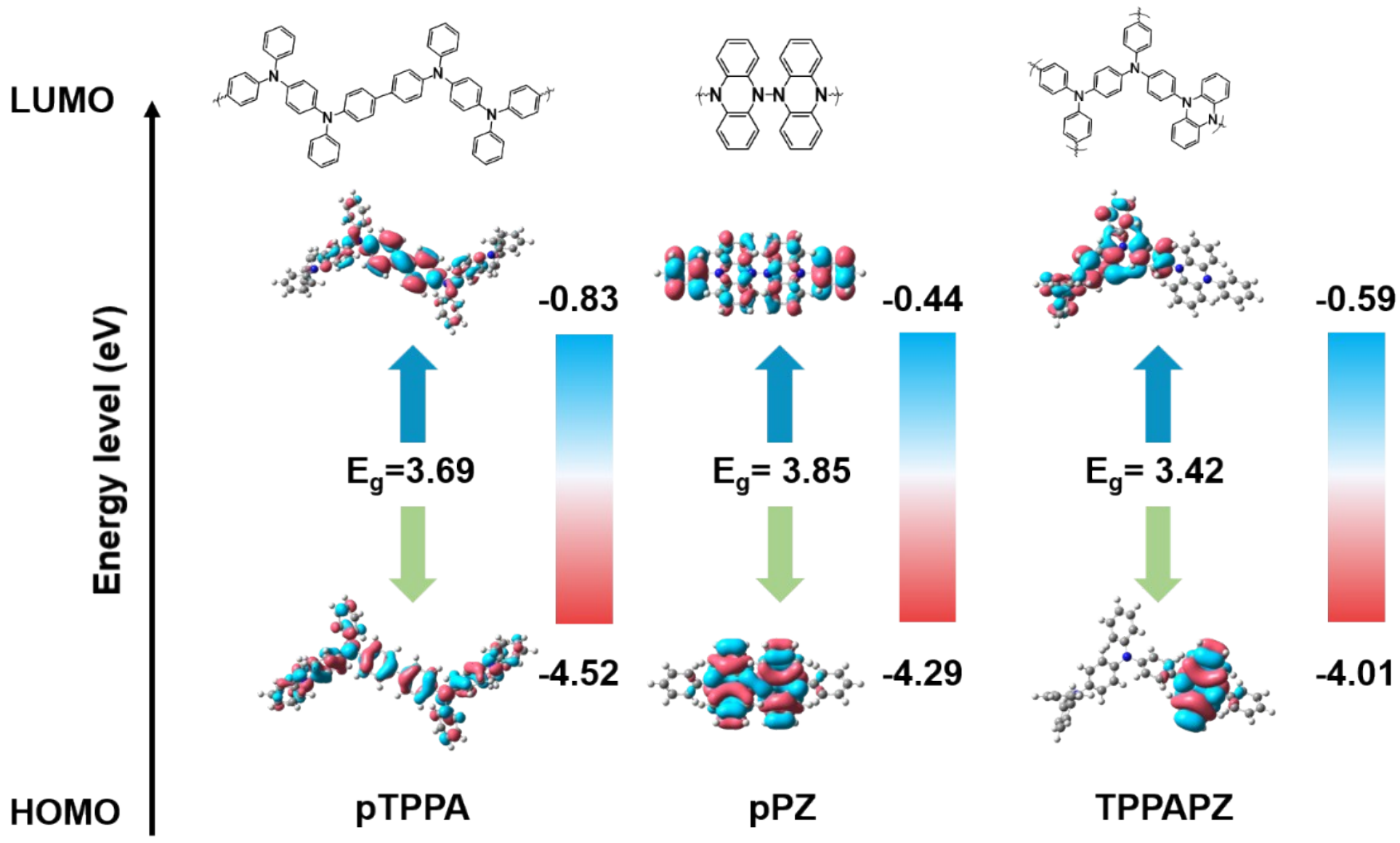

Figure S12. DFT-calculated frontier molecular orbitals and energy levels of TPPAPZ and the idealized pTPPA and pPZ polymer models, representing TPPA-TPPA, PZ-PZ and TPPA-PZ donor environments, respectively.

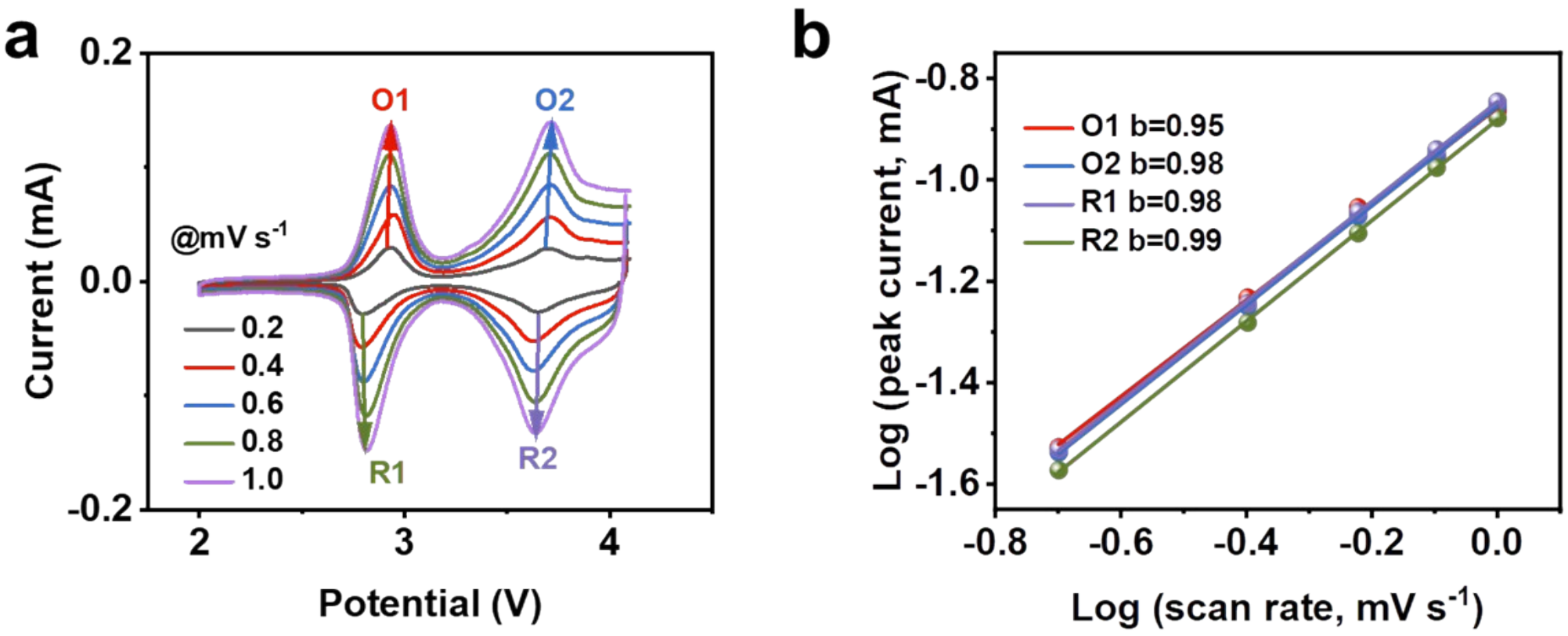

Figure S13. (a) Cyclic voltammetry (CV) curves at different scan rates; (b) Log(i_p) versus log(v) plots of redox peaks for determining b-values.

To investigate the kinetics of the redox processes in the TPPAPZ electrode, cyclic voltammetry (CV) was performed at scan rates ranging from 0.2 to 1.0 mV s^{-1} (Figure S13a). The redox peaks showed negligible potential shifts with increasing scan rate, suggesting minimal ohmic polarization and fast charge-transfer kinetics within the cell. The charge storage mechanism was further elucidated by analyzing the relationship between peak current (ip) and scan rate (v) using the power-law equation 1:[3]

$$i_p = av^b$$

The b-values, determined from log(ip) versus log(v) plots for the four characteristic peaks, were 0.95, 0.98, 0.98, and 0.99, respectively (**Figure S13b**). These values, approaching 1.0, indicate that the electrochemical behavior is primarily governed by surface-controlled pseudocapacitive processes.

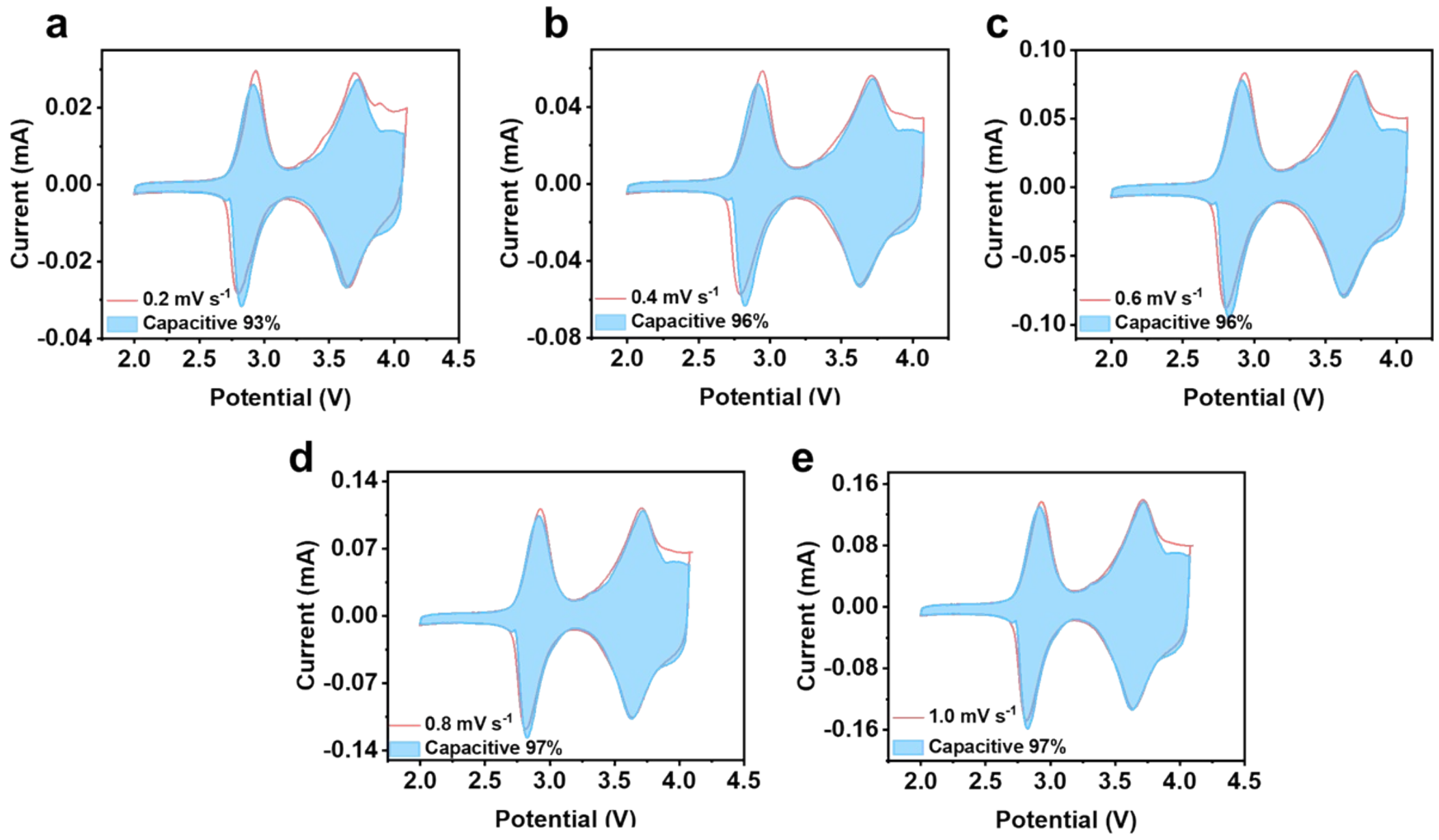

Figure S14. Contribution of capacitance control.

To quantify the relative contributions, the current was deconvoluted into capacitive (k_1v) and diffusion-controlled ($k_2v^{1/2}$) components according to Equation 2: [4]

$$i = k_1v + k_2v^{1/2}$$

As shown in **Figure S14**, the pseudocapacitive fraction increased from 93% to 97% with rising scan rate, underscoring the dominance of rapid surface-mediated ion adsorption/desorption. This kinetic feature enables efficient ion transport at high rates, thereby accounting for the superior rate performance and cycling stability of TPPAPZ based sodium-ion batteries.

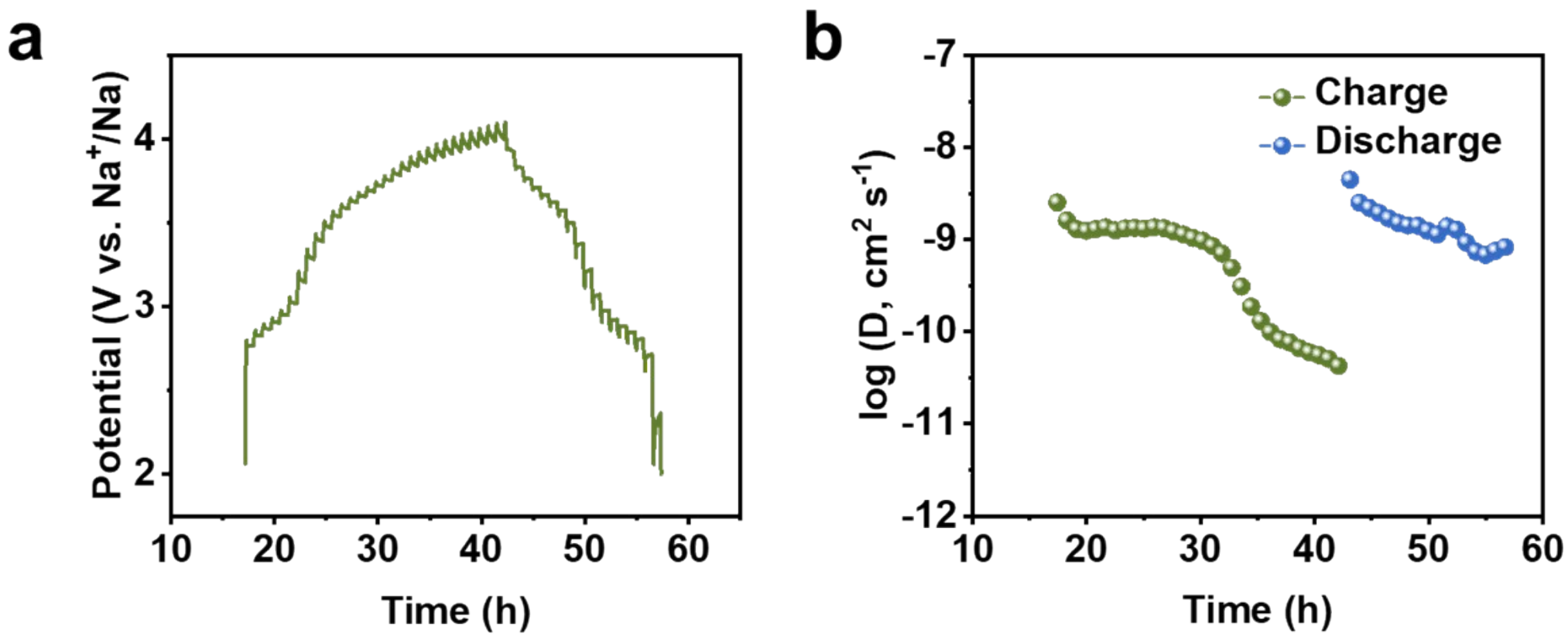

Figure S15. (a) Galvanostatic intermittent titration (GITT) curves; (b) Ionic diffusion coefficient.

The ion diffusion coefficient in the TPPAPZ electrode was assessed via the galvanostatic intermittent titration technique (GITT). Following initial activation cycles at 1.0 A g^{-1} to achieve stable capacity, GITT was conducted with current pulses of 100 mA g^{-1} for 10 min, interspersed with 40-min relaxation periods (**Figure S15a**). The calculated D values averaged ~10^{-9} cm^2 s^{-1} (**Figure S15b**), substantially higher than those typically reported for organic sodium-ion battery electrodes (~10^{-12} cm^2 s^{-1}).[5-7] This enhanced diffusivity further explains the excellent high-rate capability of the material.

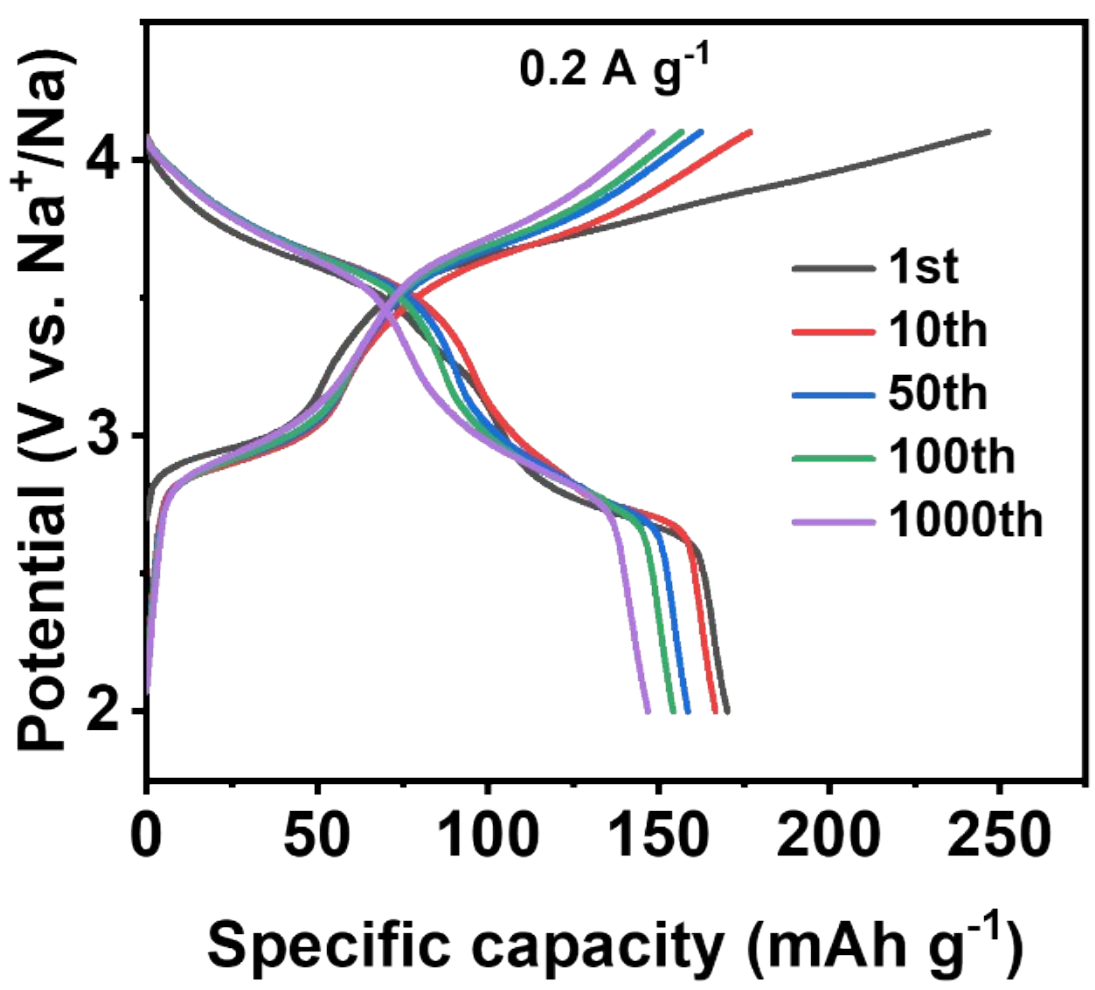

Figure S16. Charge-discharge curves of the TPPAPZ electrode at a current density of 0.2 A g^{-1}.

Table S1. Electrochemical performance of organic polymeric sodium-ion cathodes.

Cathode	Potential (V vs. Na^+/Na)	Current density ($A g^{-1}$)	number of cycles	Specific Capacity ($mAh g^{-1}$)	capacity retention ratio	Ref.
p-CZPDPZ	3.2	1	2500	57	38%	8
TAQ	2	0.2	2000	220	92%	9
PZ-HATN	2.6	10	3000	106	71%	10
TPAD	3	1	2000	75	75%	5
PVDMP	3.2	1	4000	150	65%	11
PTCDI-DAQ	2	3	1000	131	72%	12
TPyPz	3.1	2	1000	75	75%	13
BPyPz	3.1	2	1000	140	90%	13
DAAQ-HCCP	1	2	1000	75	90%	14
TPPAPZ	3.4	10	1000<br>10000<br>50000	129<br>126<br>108	96%<br>93%<br>80%	This work

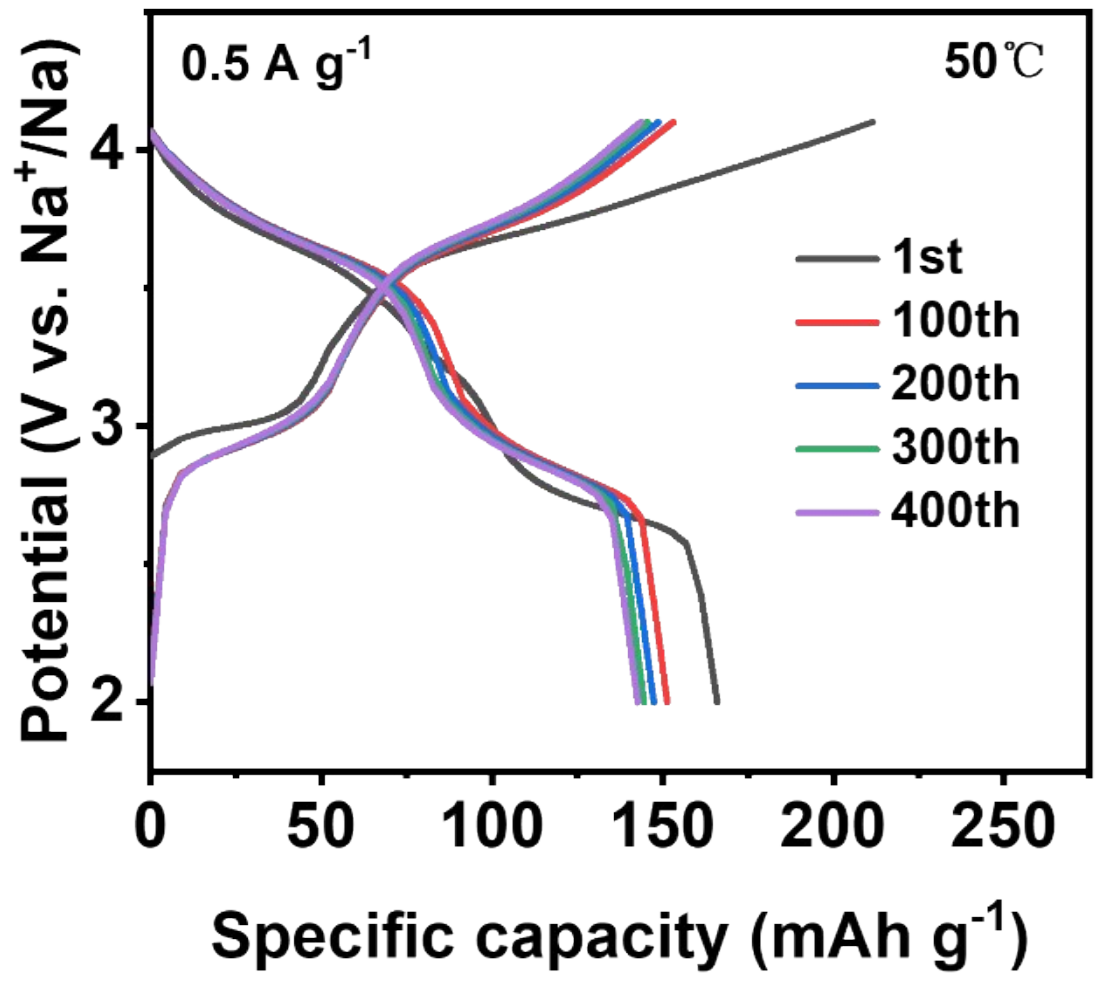

Figure S17. Charge–discharge curves of the TPPAPZ electrode at a current density of 0.5 A g^{-1} and 50 °C.

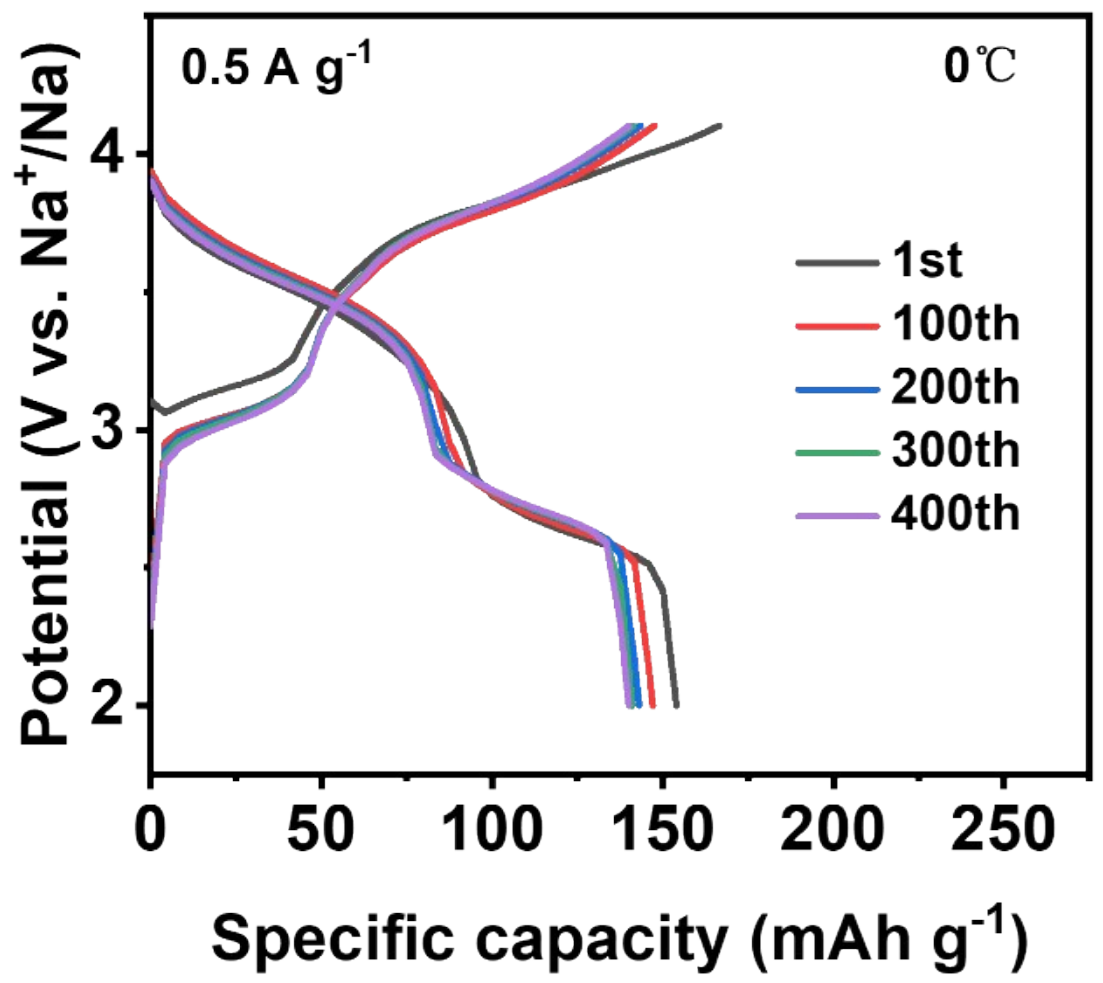

Figure S18. Charge–discharge curves of the TPPAPZ electrode at a current density of 0.5 A g^{-1} and 0 °C.

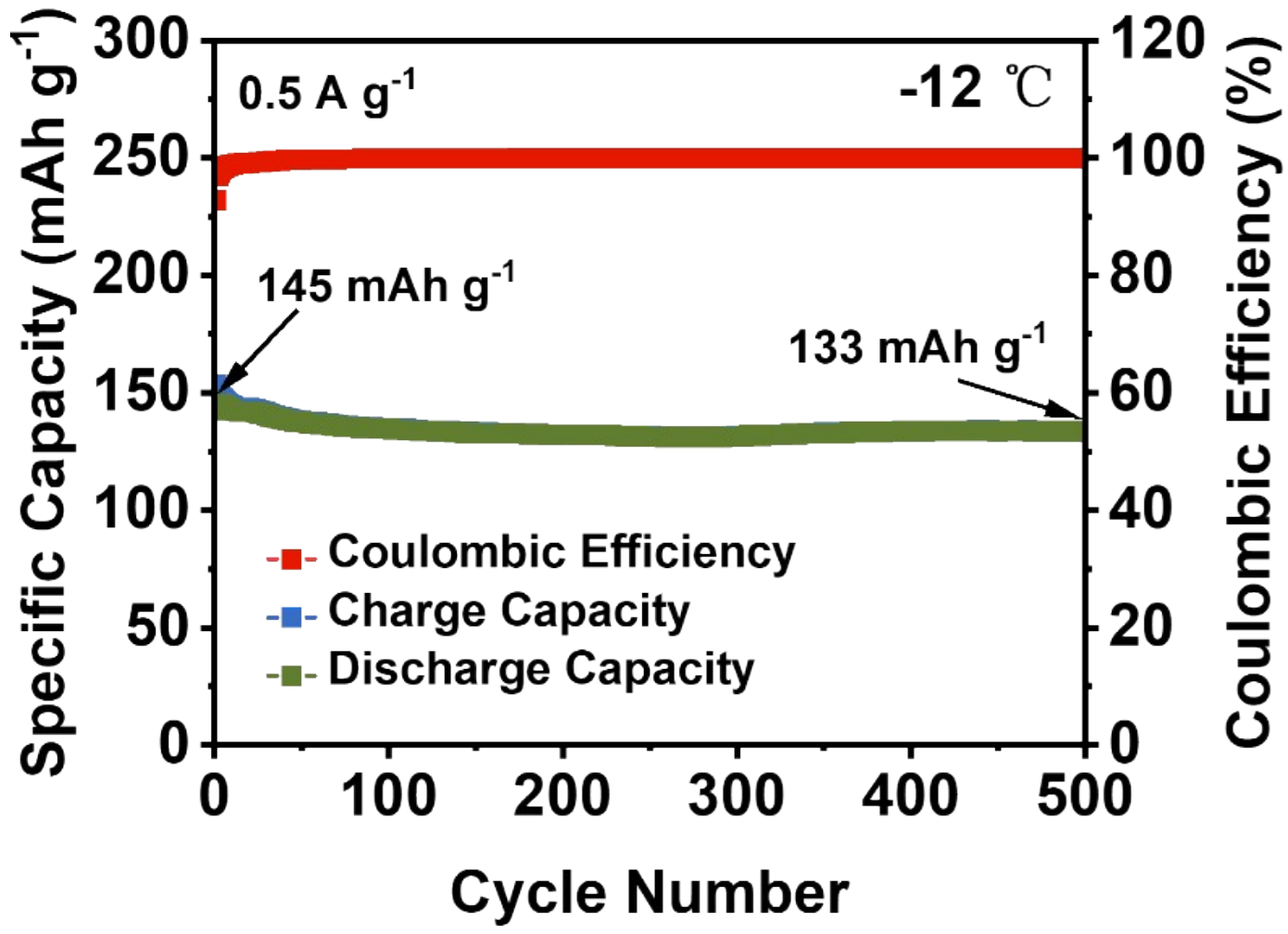

Figure S19. Long-term cycling performance at -12 °C and 0.5 A g^{-1}.

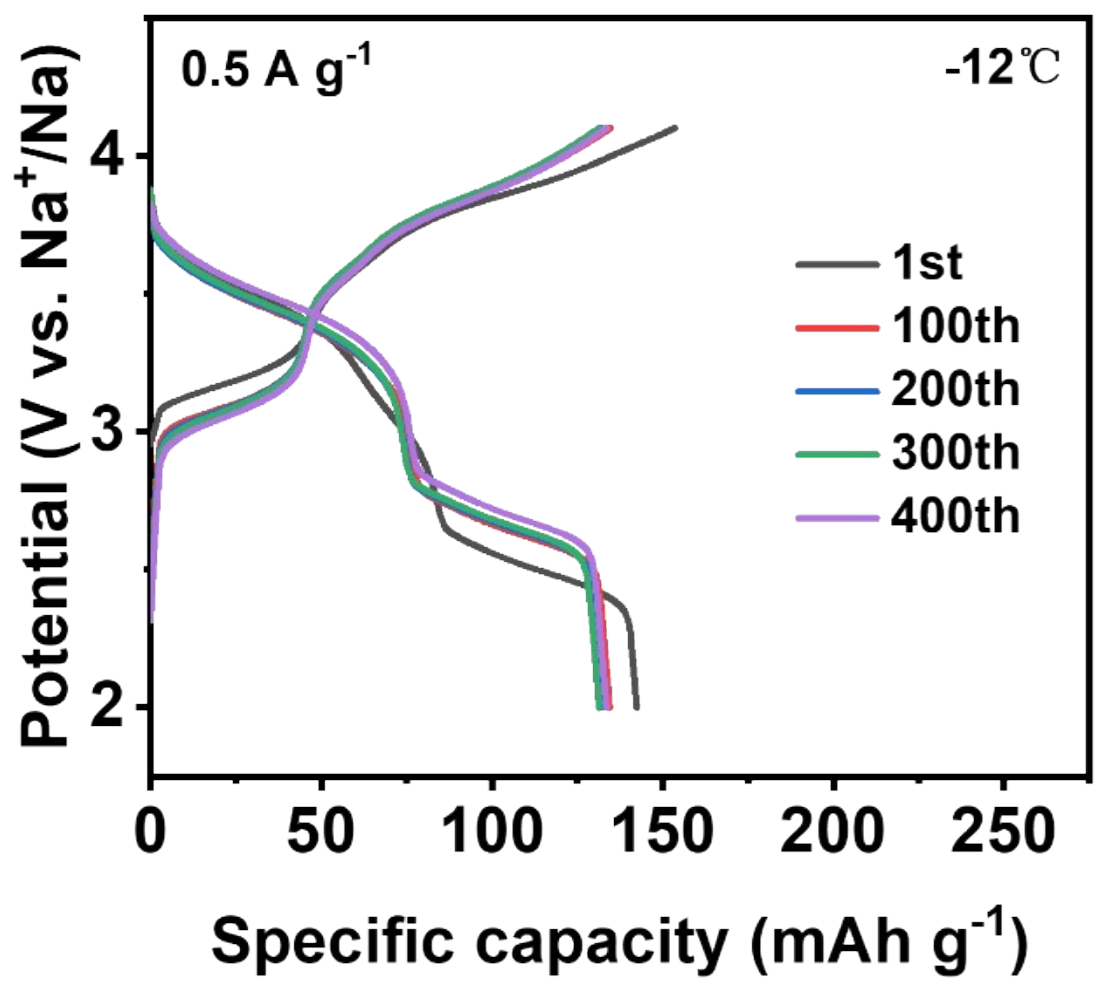

Figure S20. Charge-discharge curves of the TPPAPZ electrode at a current density of 0.5 A g^{-1} and -12 °C.

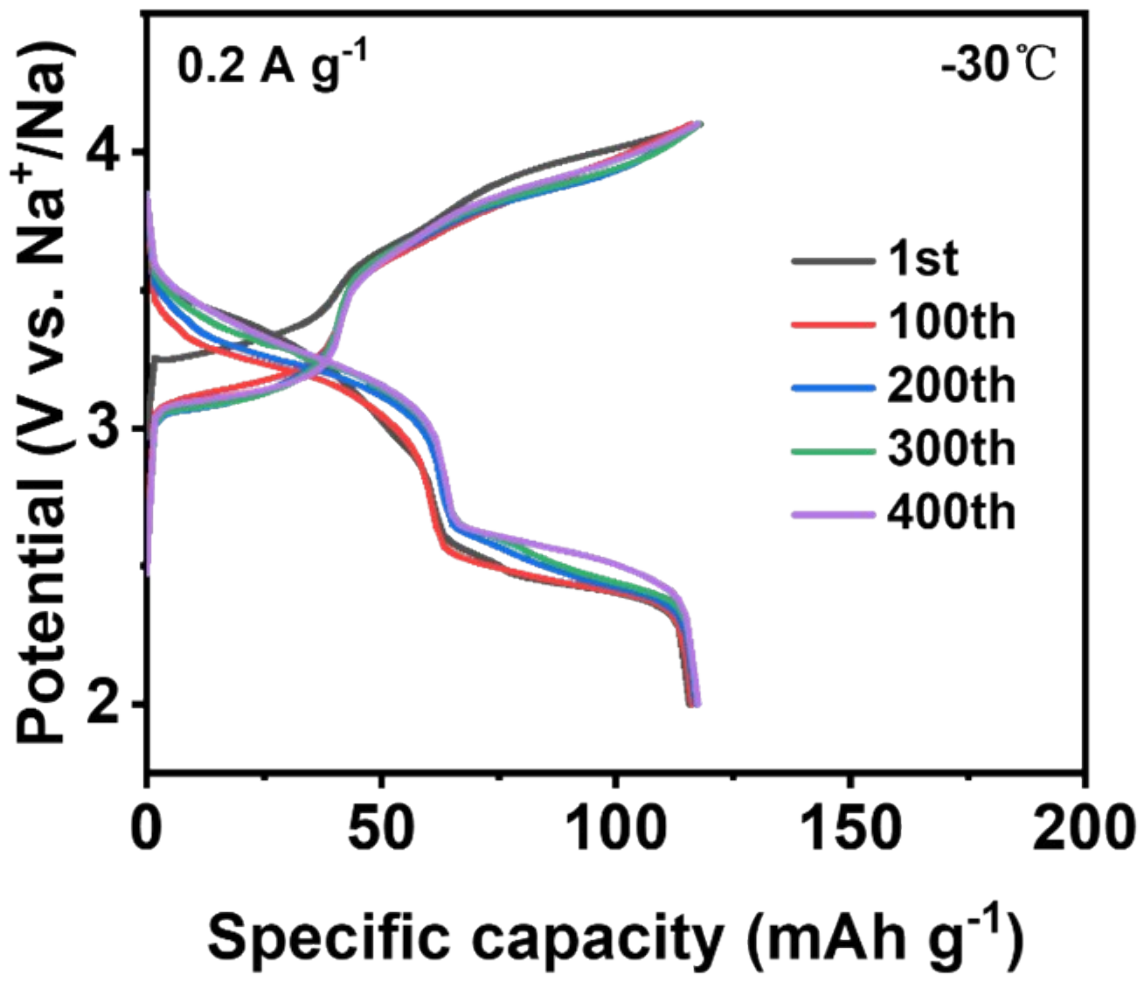

Figure S21. Charge-discharge curves of the TPPAPZ electrode at a current density of 0.2 A g^{-1} and -30 °C.

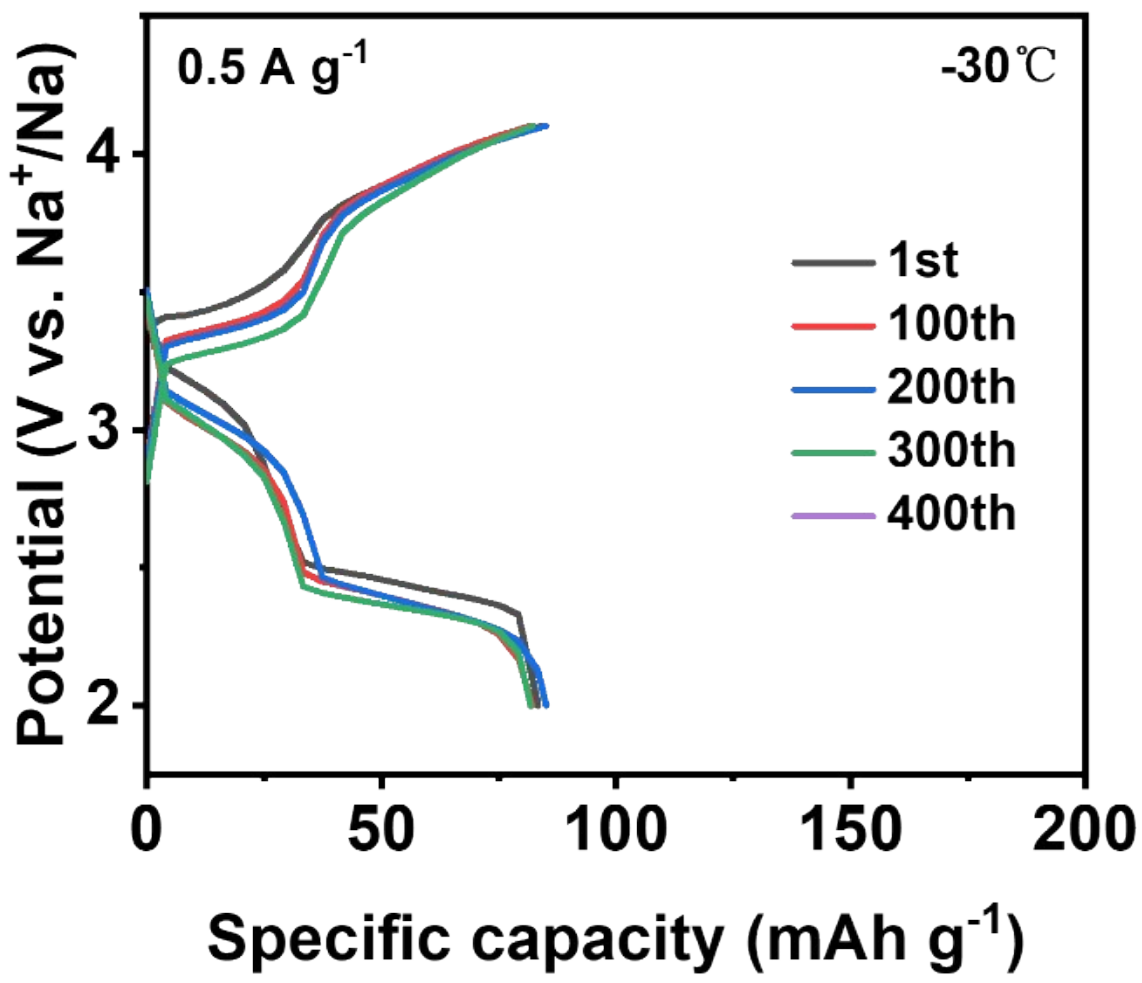

Figure S22. Charge-discharge curves of the TPPAPZ electrode at a current density of 0.5 A g^{-1} and -30 °C.

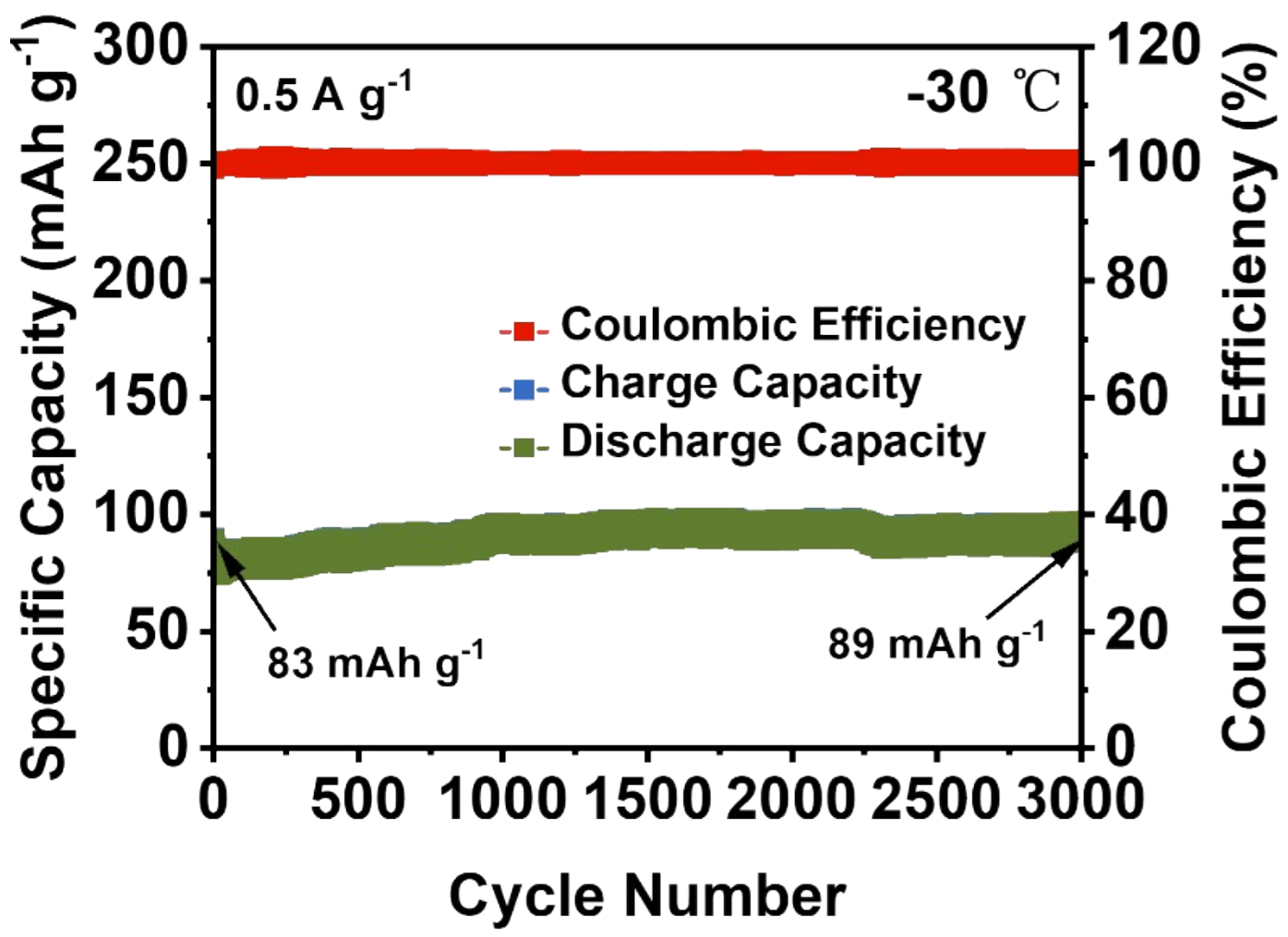

Figure S23. Long-term cycling performance at -30 °C and 0.5 A g^{-1}.

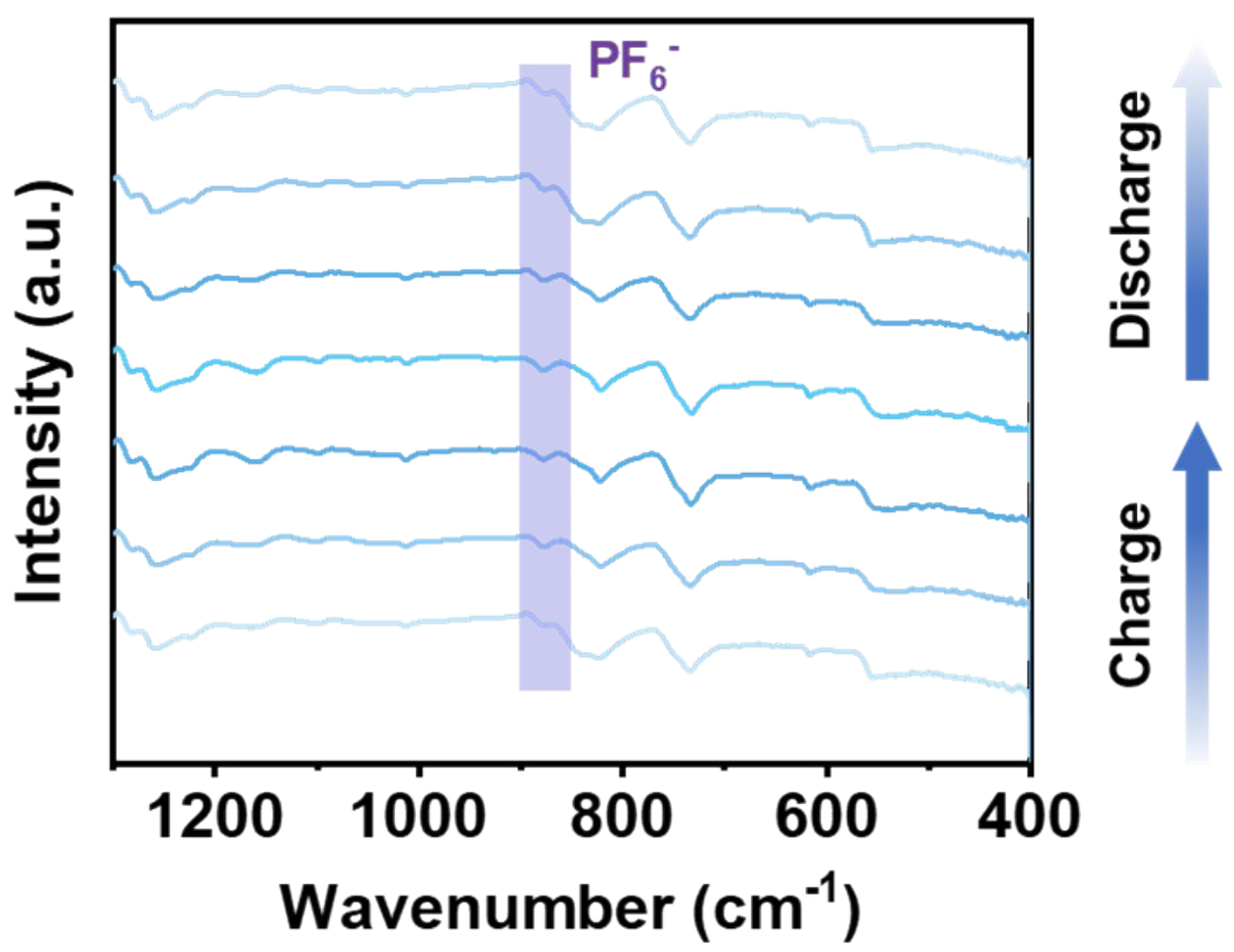

Figure S24. *Ex-situ* FTIR spectra for the TPPAPZ cathode during the charge and discharge processes

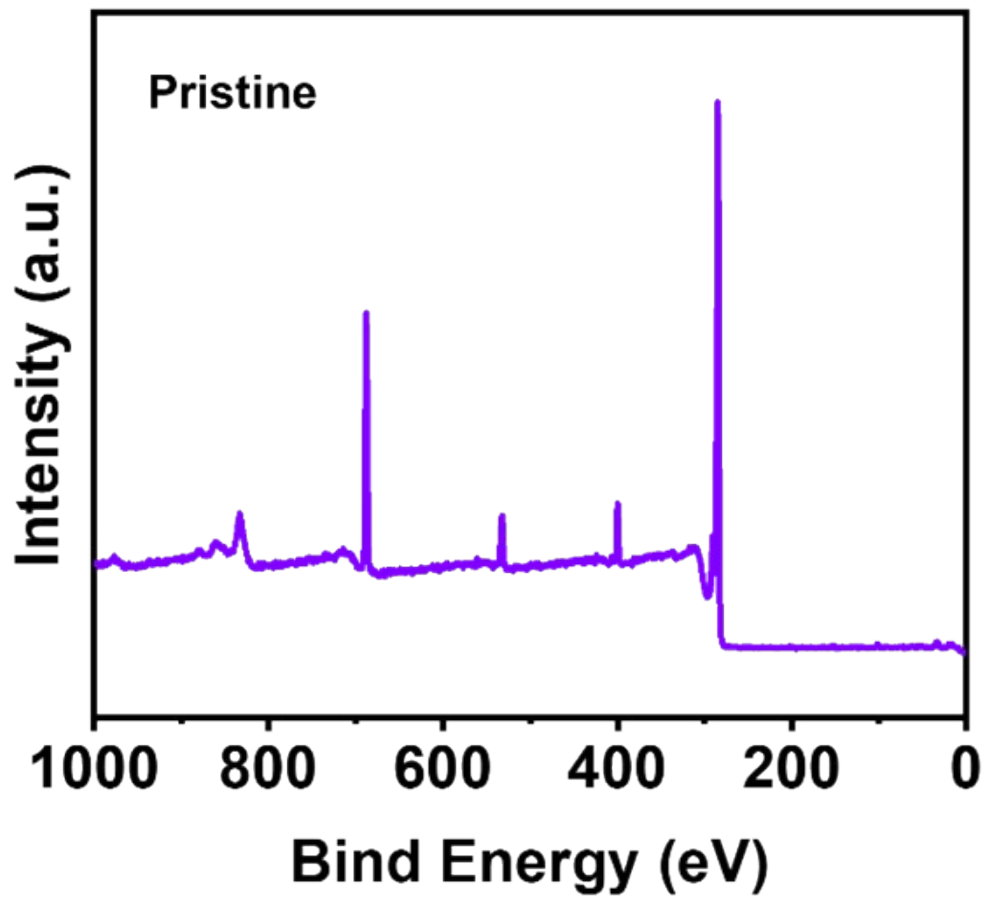

Figure S25. XPS survey spectrum of TPPAPZ electrode in the pristine state.

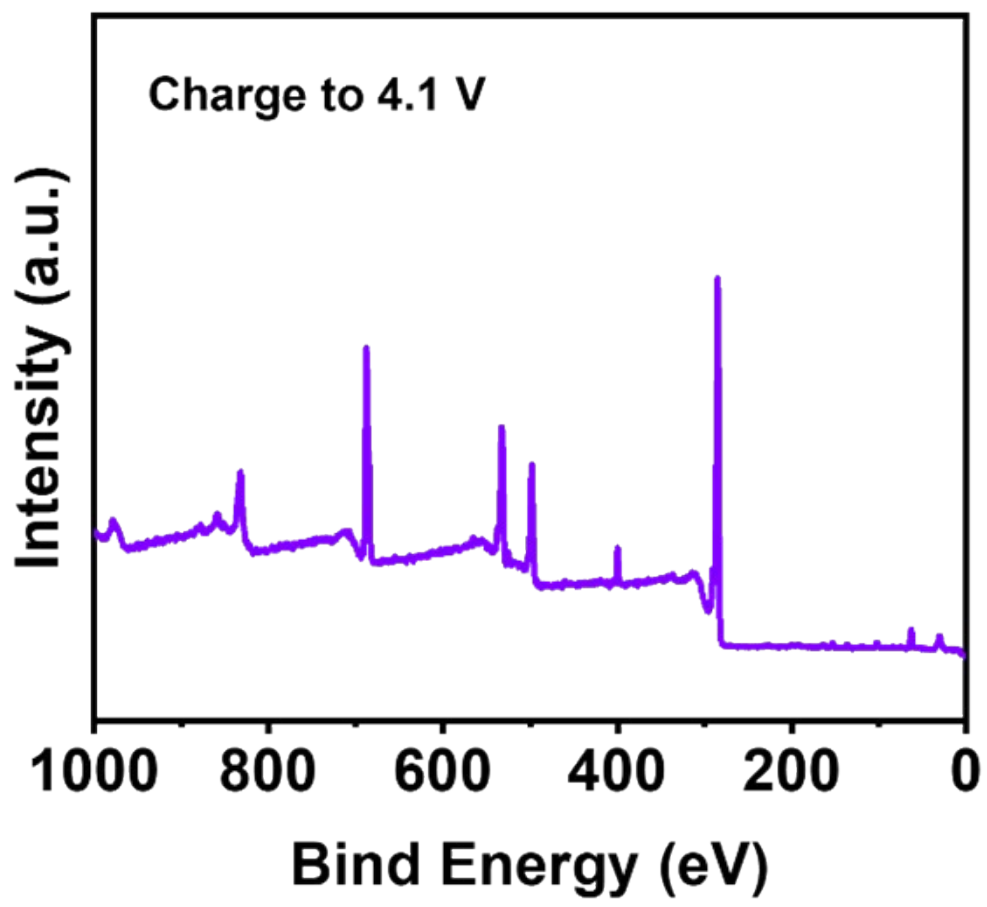

Figure S26. XPS survey spectrum of TPPAPZ electrode in a fully charged state.

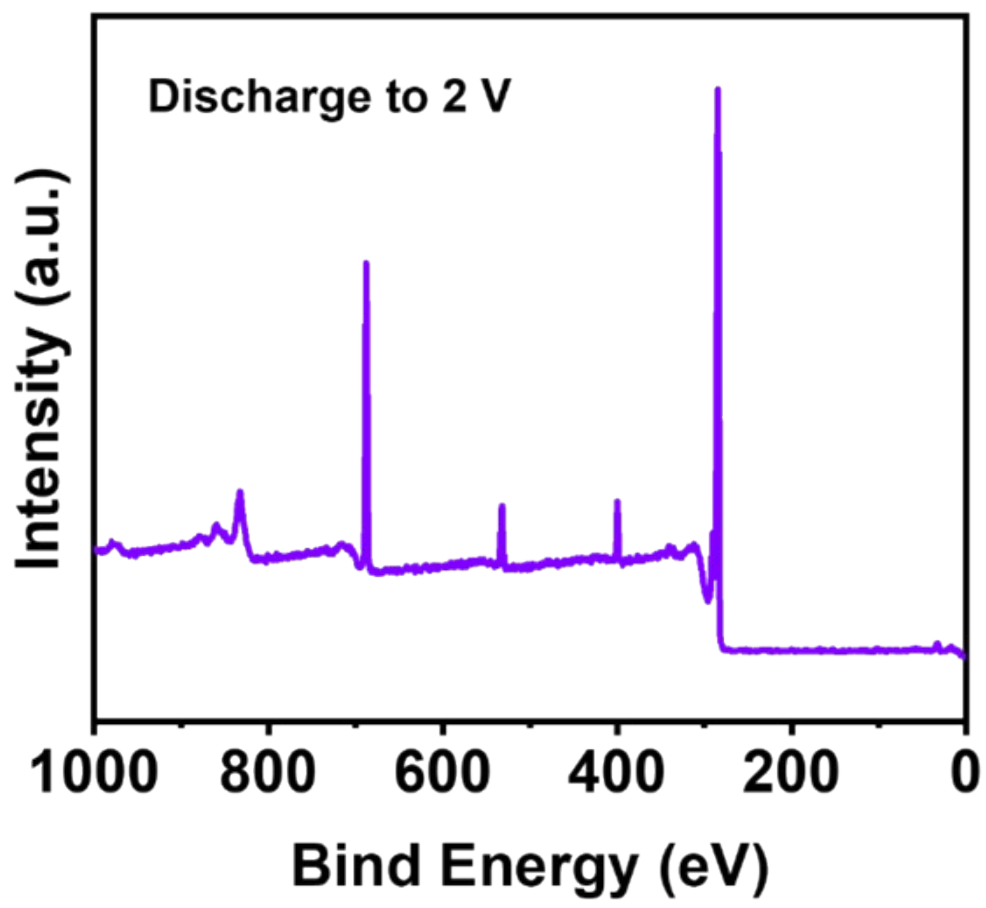

Figure S27. XPS survey spectrum of TPPAPZ electrode in a fully discharged state.

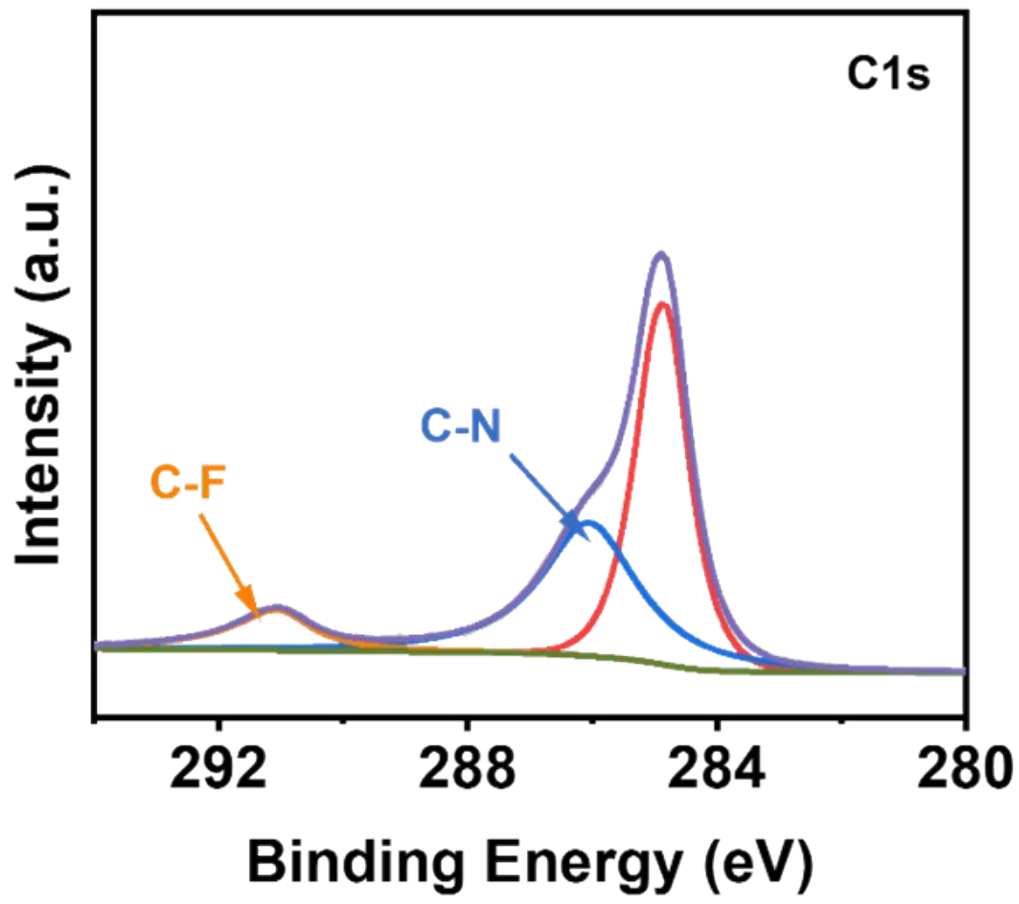

Figure S28. C 1s XPS spectrum of TPPAPZ electrode in the pristine state.

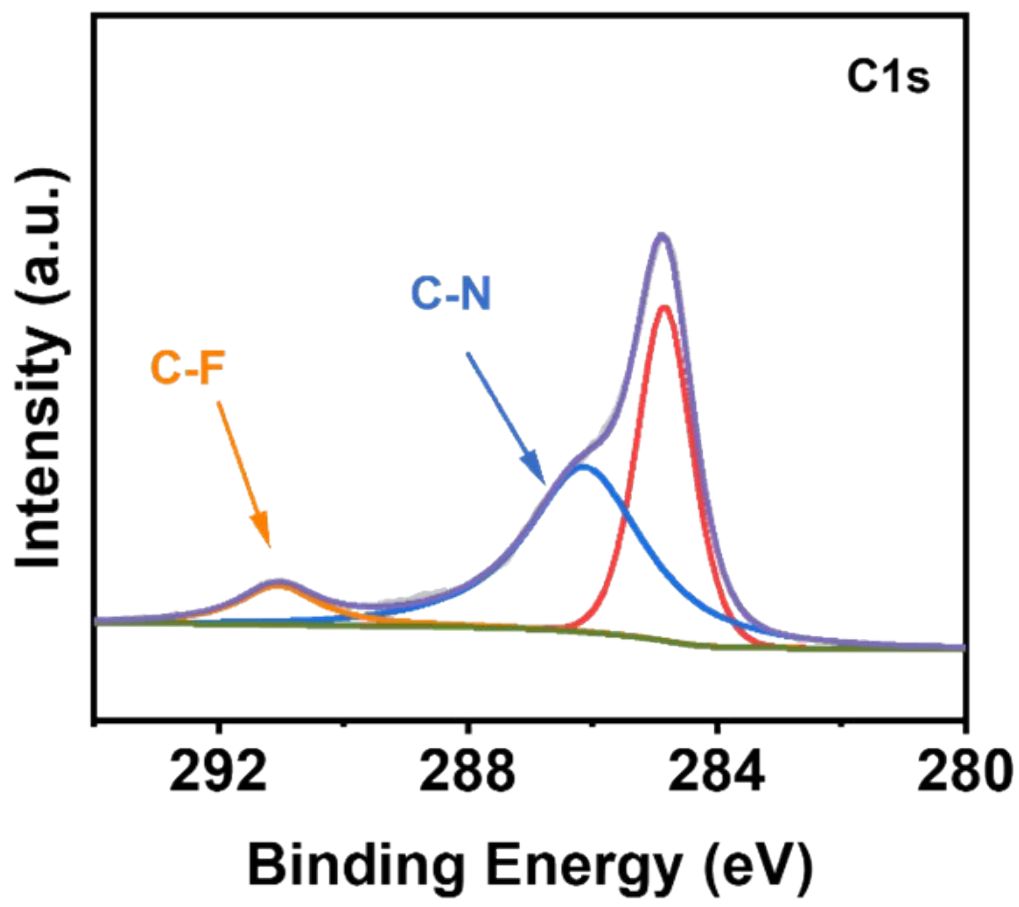

Figure S29. C 1s XPS spectrum of TPPAPZ electrode in a fully charged state.

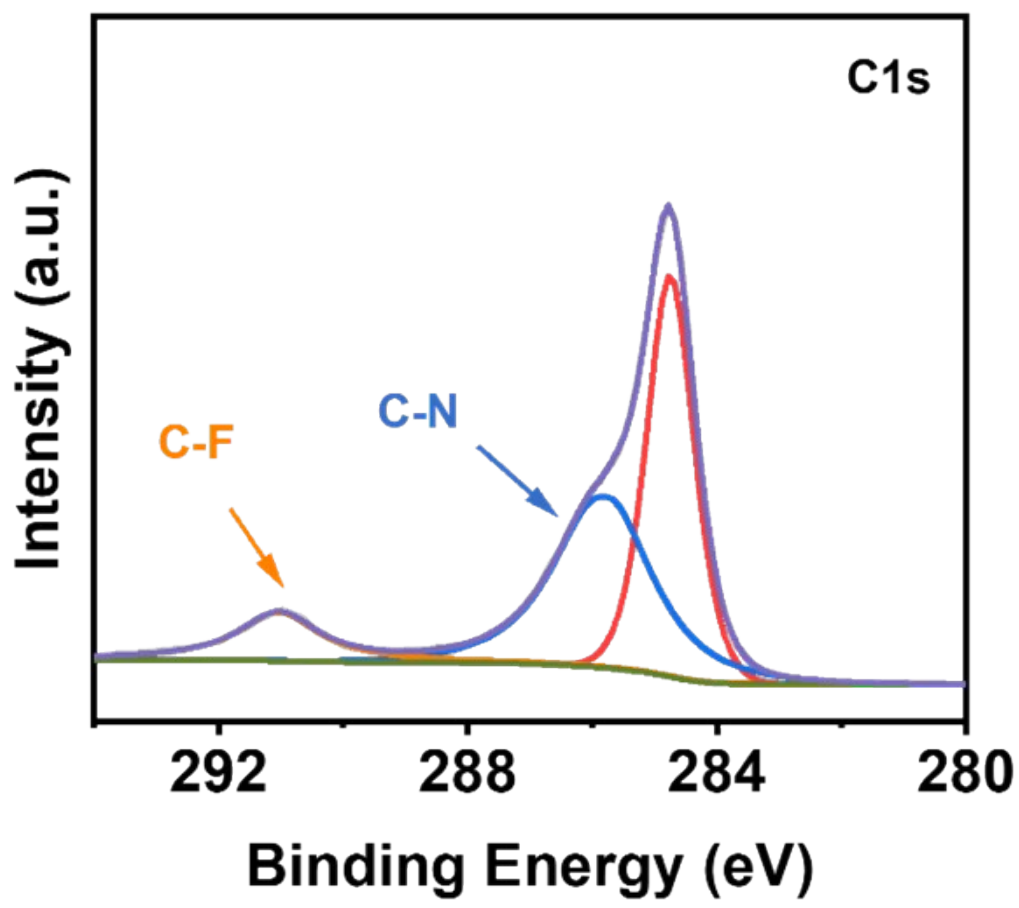

Figure S30. C 1s XPS spectrum of TPPAPZ electrode in a fully discharged state.

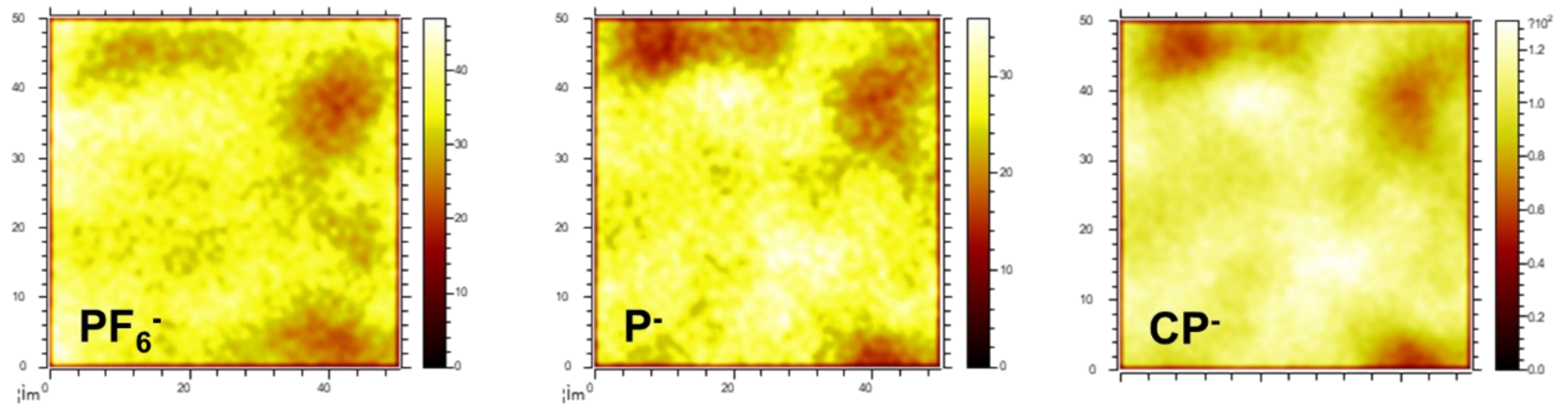

Figure S31. 2D render of composition and concentration distribution of the full charge electrode.

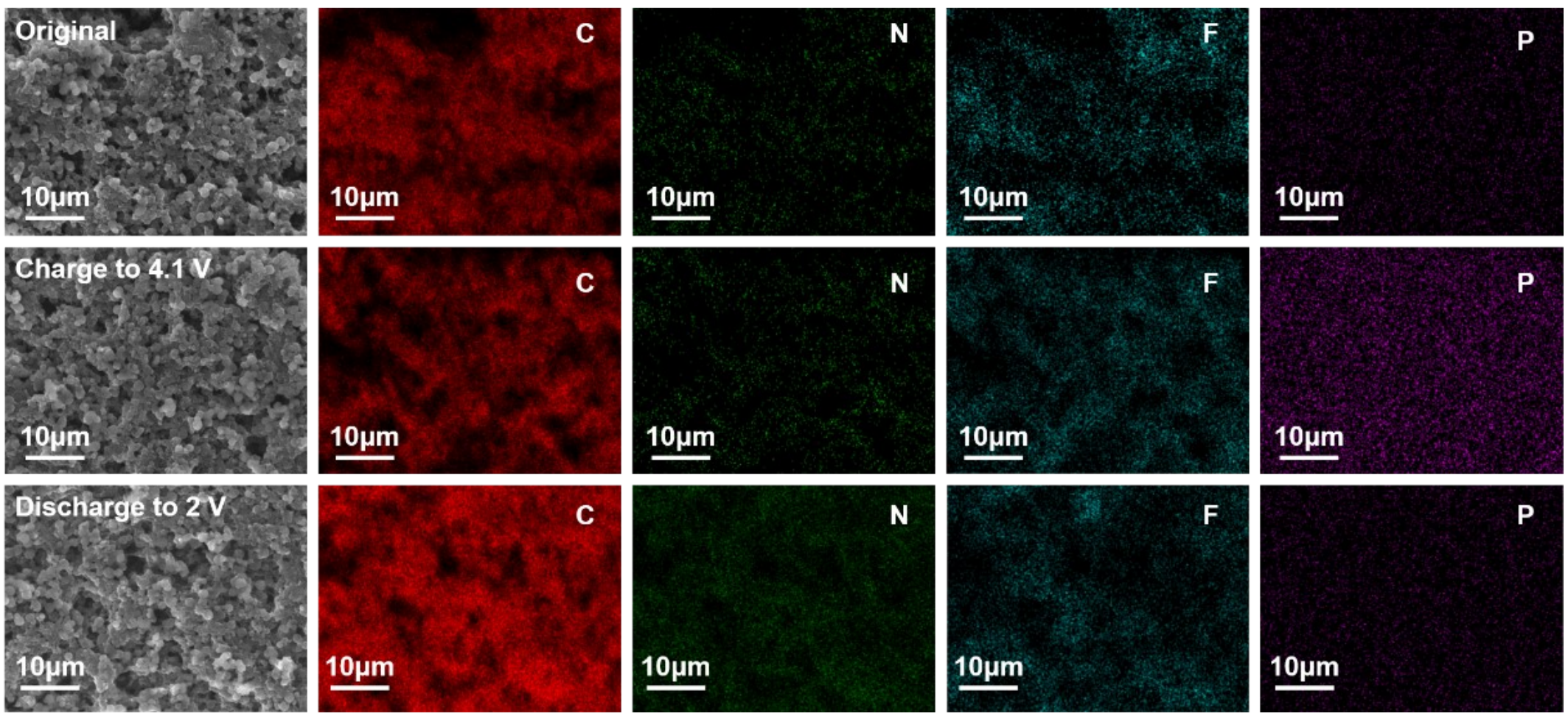

Figure S32. SEM image and mapping at different charge and discharge stages.

Table S2. Element distribution of TPPAPZ electrode in initial state

Element	Wt%	Wt% Sigma	At %
C	85.09	0.43	87.81
N	10.72	0.45	9.49
F	4.06	0.08	2.65
P	0.13	0.02	0.05

Table S3. Element distribution of TPPAPZ electrode in charge to 4.1 V.

Element	Wt%	Wt% Sigma	At %
C	76.95	0.37	82.00
N	11.86	0.41	10.84
F	9.71	0.11	6.54
P	1.48	0.03	0.61

Table S4. Element distribution of TPPAPZ electrode in discharge to 2 V.

Element	Wt%	Wt% Sigma	At %
C	83.59	0.35	86.62
N	11.42	0.37	10.14
F	4.87	0.07	3.19
P	0.13	0.01	0.05

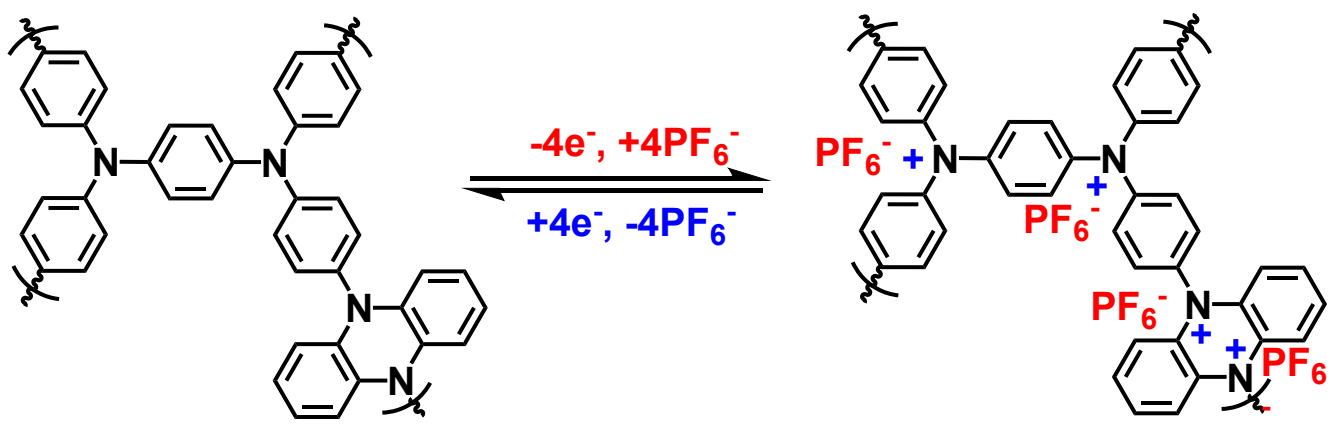

Figure S33. The charging and discharging mechanism of TPPAPZ.

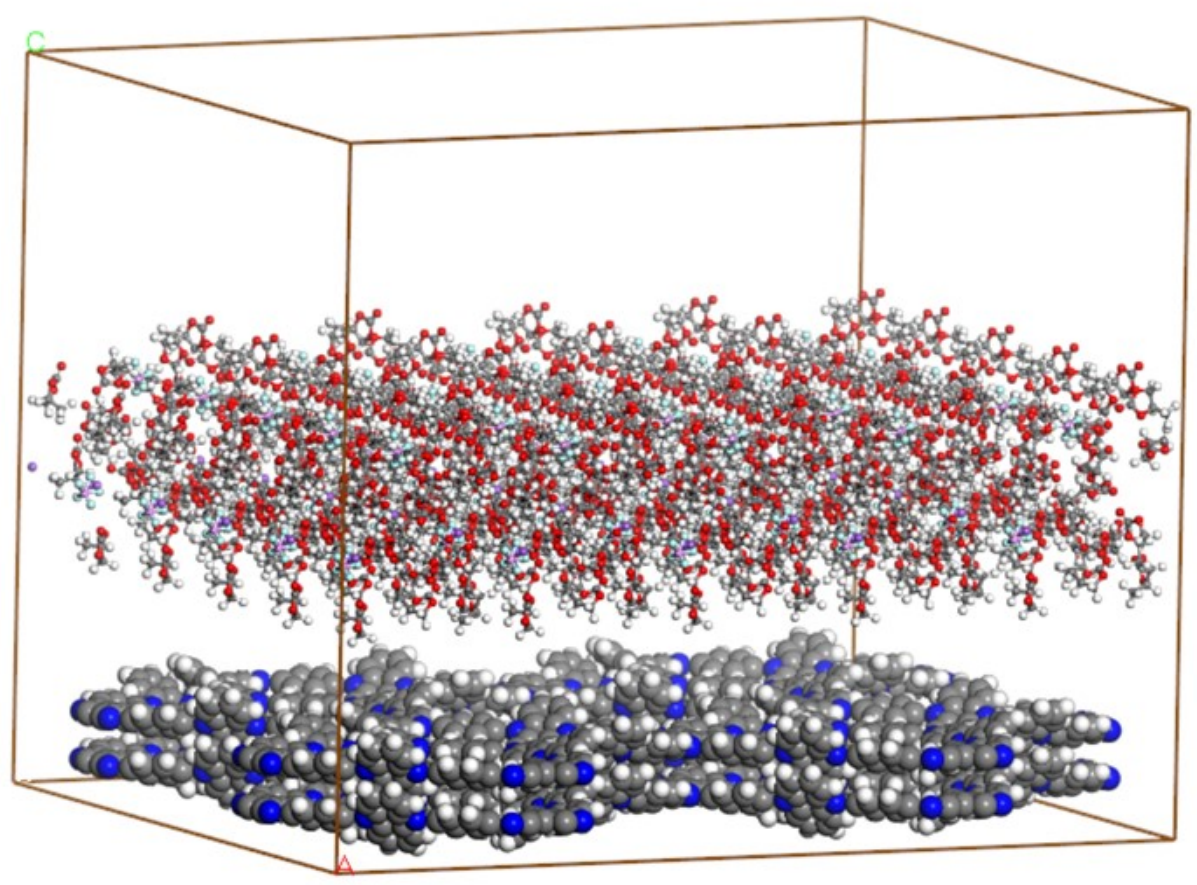

Figure S34. Simulation model of TPPAPZ and electrolyte.

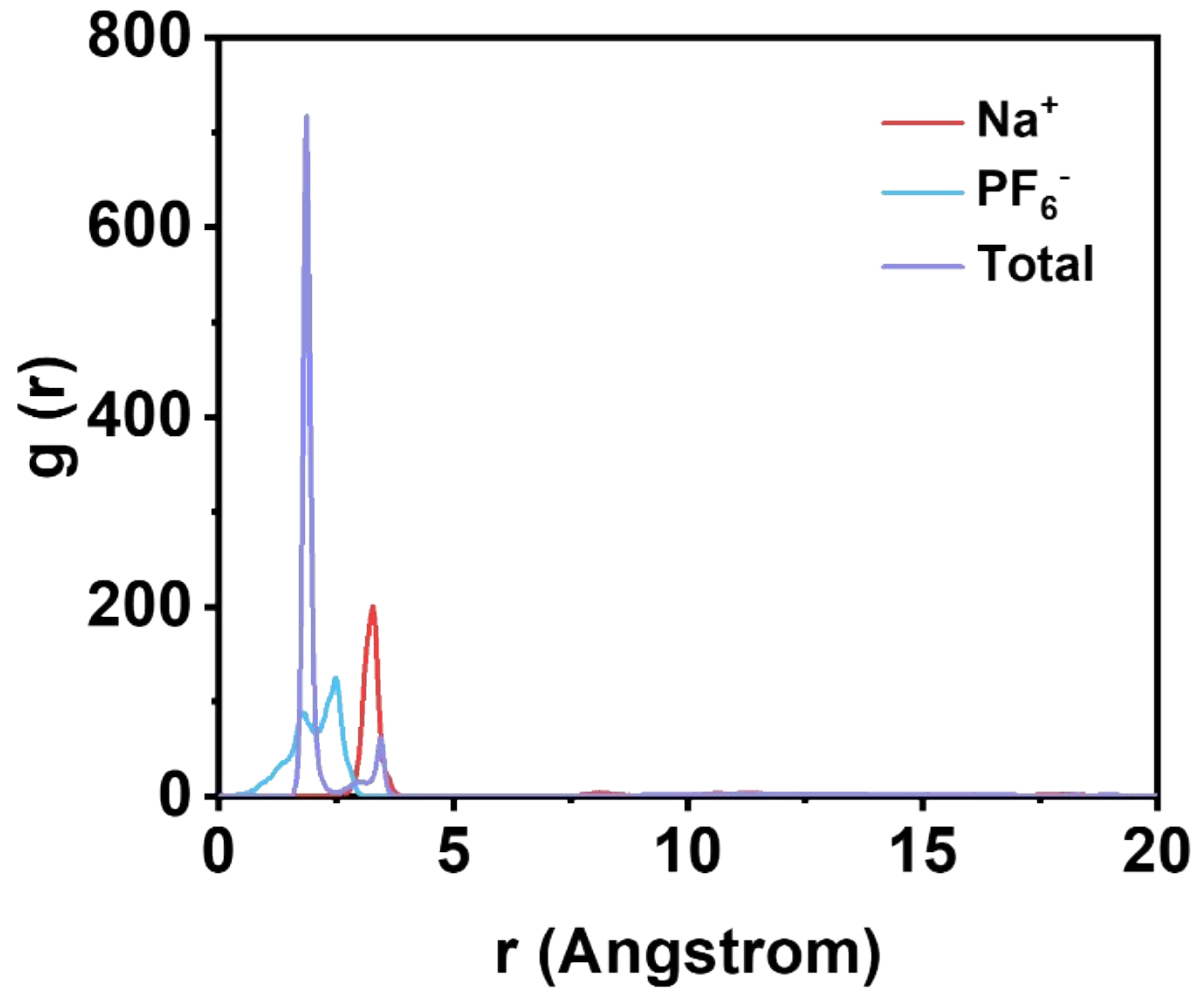

Figure S35. Radial distribution function (RDF) curves.

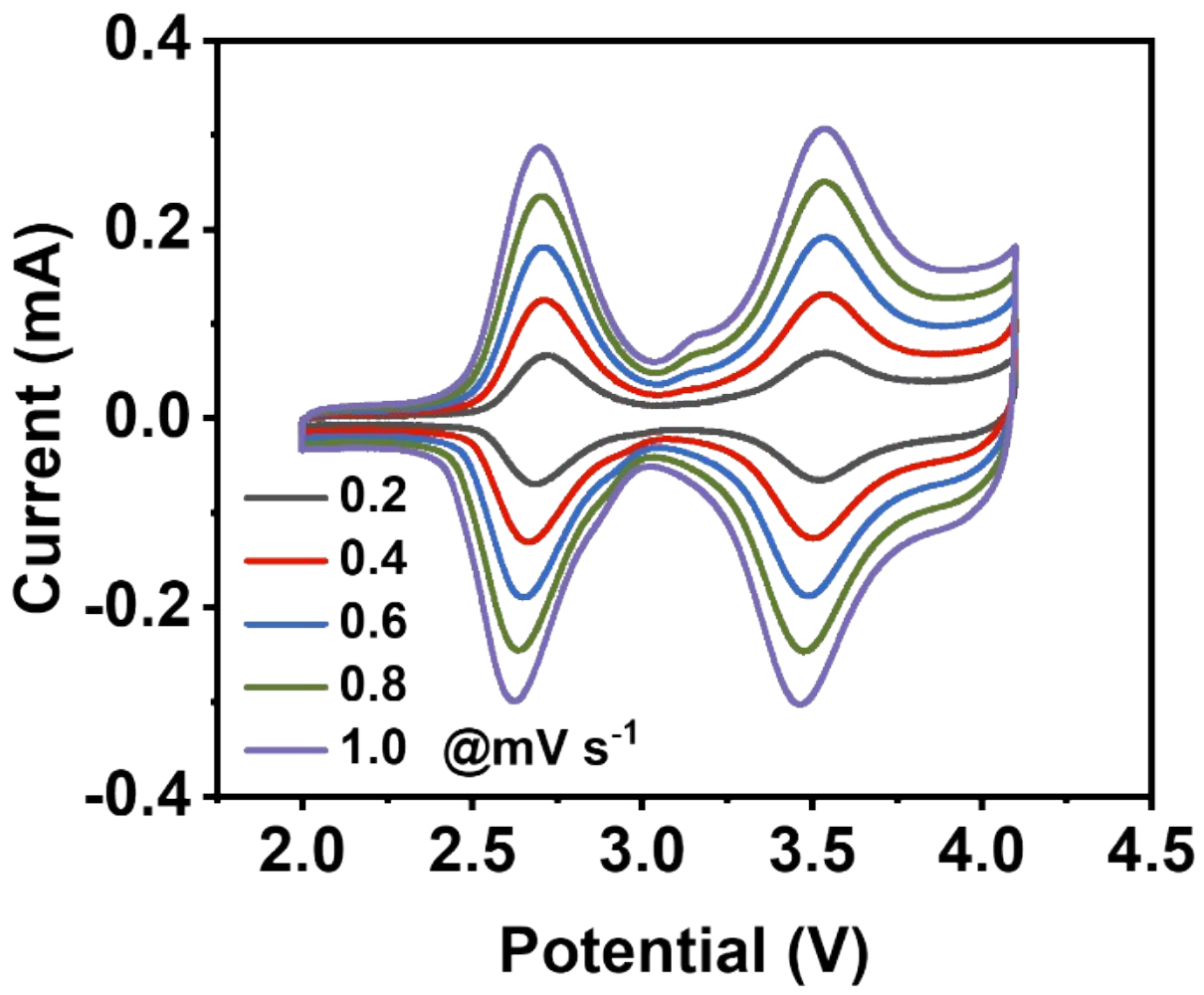

Figure S36. Cyclic voltammetry (CV) curves at different scan rates.

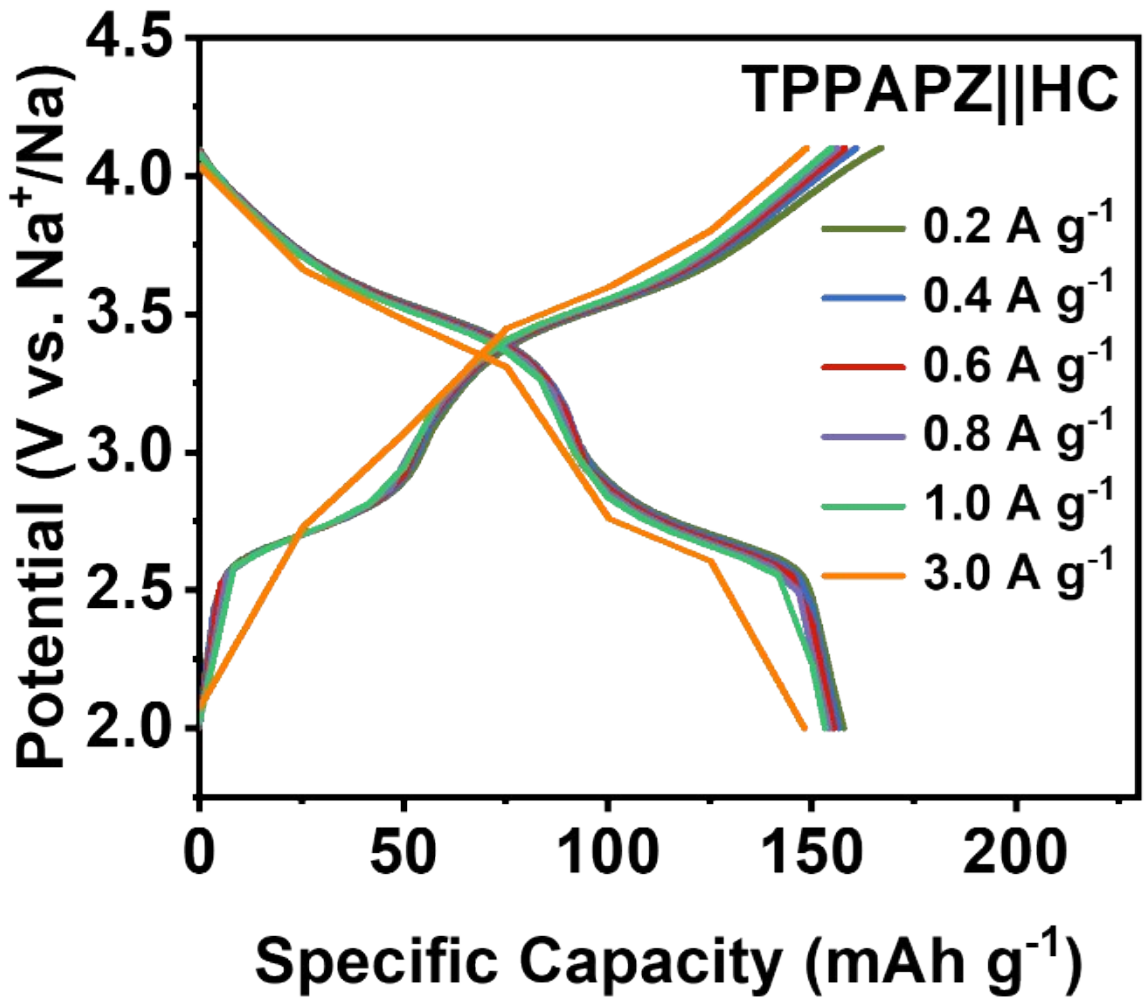

Figure S37. Charge-discharge profiles at various current densities

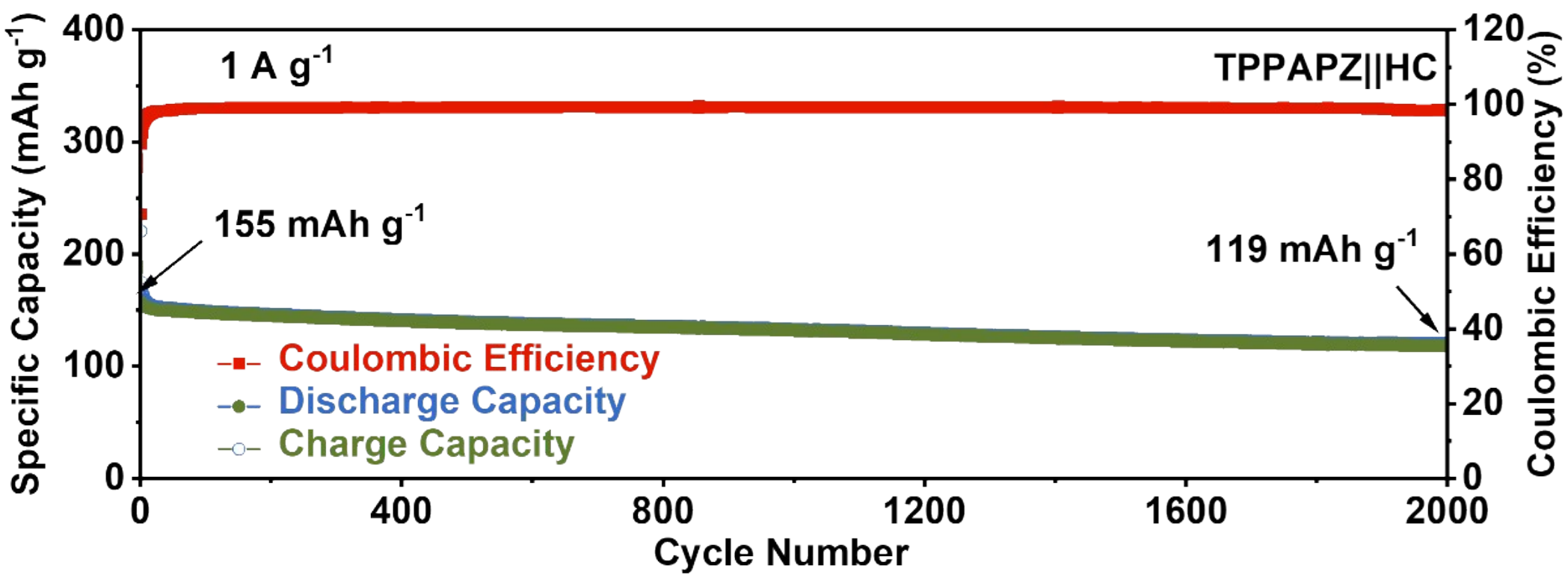

Figure S38. Long-term cycling performance of the TPPAPZ||HC full cell at 1 A g^{-1}.

References

1. D. C. Mayer, A. Manzi, R. Medishetty, B. Winkler, C. Schneider, G. Kieslich, A. Pöthig, J. Feldmann and R. A. Fischer, J. Am. Chem. Soc., 2019, 141, 11594-11602.
2. L. Hou, X. Jing, H. Huang and C. Duan, Chem. Commun., 2023, 59, 3407-3410.
3. J. Duan, L. Teng, H. Liu, X. Zhang, H. Yu, Q. Huang, Y. Li, M. Liu, H. Hu and W. Lyu, Angew. Chem. In. Ed., 2025, 137, e202517853.
4. X. Yang, L. Gong, Y. Jin, T. Zheng, X. Wang, Q. Zhi, B. Yu, K. Wang and J. Jiang, CCS Chemistry, 2025, 1-13.
5. L. Cheng, X. Yan, J. Yu, X. Zhang, H. G. Wang, F. Cui and Y. Wang, Adv. Mater., 2025, 37, 2411625.
6. M. Zhang, R. Zhou, Y. Qin, X. Zhong, Q. Liu, X. Han, F. Zhang, X. Ou, J. Han and C.-S. Lee, Energy Storage Materials, 2025, 74, 103879.
7. S. Zhang, J. Cai, H. Li, F. Xing, L. Chen, X. Wang and X. He, Adv. Energy Mater., 2025, 15, 2403029.
8. W. Li, X. Li, Y. Gu, B. Zhu, Y. Zheng, J. Shi and W. Tang, Mater. Today, 2025, 88, 229.
9. T. Chen, J. Wang, B. Tan, K. J. Zhang, H. Banda, Y. Zhang, D.-H. Kim and M. Dinca, J. Am. Chem. Soc., 2025, 147, 6181-6192.
10. W. Hu, W. Zhang, A. Yu, C. Li, S. Mei and C.-J. Yao, Energy Storage Mater., 2025, 75, 104011.
11. A. Duan, Z. Wang, X. Huang and Y. Li, Angew. Chem. In. Ed., 2023, 62, e202302754.
12. F. Yu, W. Tang, S. Wang, M. Guo, W. Deng, J. Hu, S. Jia and C. Fan, Adv. Funct. Mater., 2023, 33, 2300740.
13. J. Zhang, H. Liu, K. Jia, X. Li, X. Liu, L. Zhu, R. He and F. Wu, J. Mater. Chem. A, 2023, 11, 2711-2717.
14. M.-M. Hu, H. Huang, Q. Gao, Y. Tang, Y. Luo, Y. Deng and L. Zhang, Energy Fuel., 2020, 35, 1851-1858.
